# Supplementary material for: Highly conductive single-molecule junctions through electrocatalytic formation of benzyl-type Au‒C bonds
Source: Nat Commun. 2025 Aug 18;16:7692. doi: 10.1038/s41467-025-62961-x (PMC12361500; doi:10.1038/s41467-025-62961-x)
Supplement: Supplementary file 1 — Supplementary Information [file 41467_2025_62961_MOESM1_ESM.pdf]

Supplementary information

Highly conductive single-molecule junctions through electrocatalytic formation of benzyl-type Au–C bonds

Yaxuan Zhang[1,2,†], Kai Qu[1,2,†], Ting Pan[1,2,†], Yaqi Zhang[1,2], Leng Wang[1,2] & Hongliang Chen[1,2,3,✉]

[1]Stoddart Institute of Molecular Science, Department of Chemistry, Zhejiang University, Hangzhou 310058, P. R. China

[2]ZJU-Hangzhou Global Scientific and Technological Innovation Center, Zhejiang University, Hangzhou 311215, P. R. China

[3]Beijing National Laboratory for Molecular Sciences, Beijing 100871, P. R. China

[†]These authors contributed equally: Y. Zhang, K. Qu, T. Pan

✉e-mail: hongliang.chen@zju.edu.cn

Table of Contents

Supplementary Note 1. Synthetic protocols 3

Supplementary Note 2. NMR spectroscopy 13

Supplementary Note 3. Electrical data 26

Supplementary Note 4. Characterization of radicals 37

Supplementary Note 5. Theoretical calculations 39

Supplemental references 43

Supplementary Note 1. Synthetic protocols

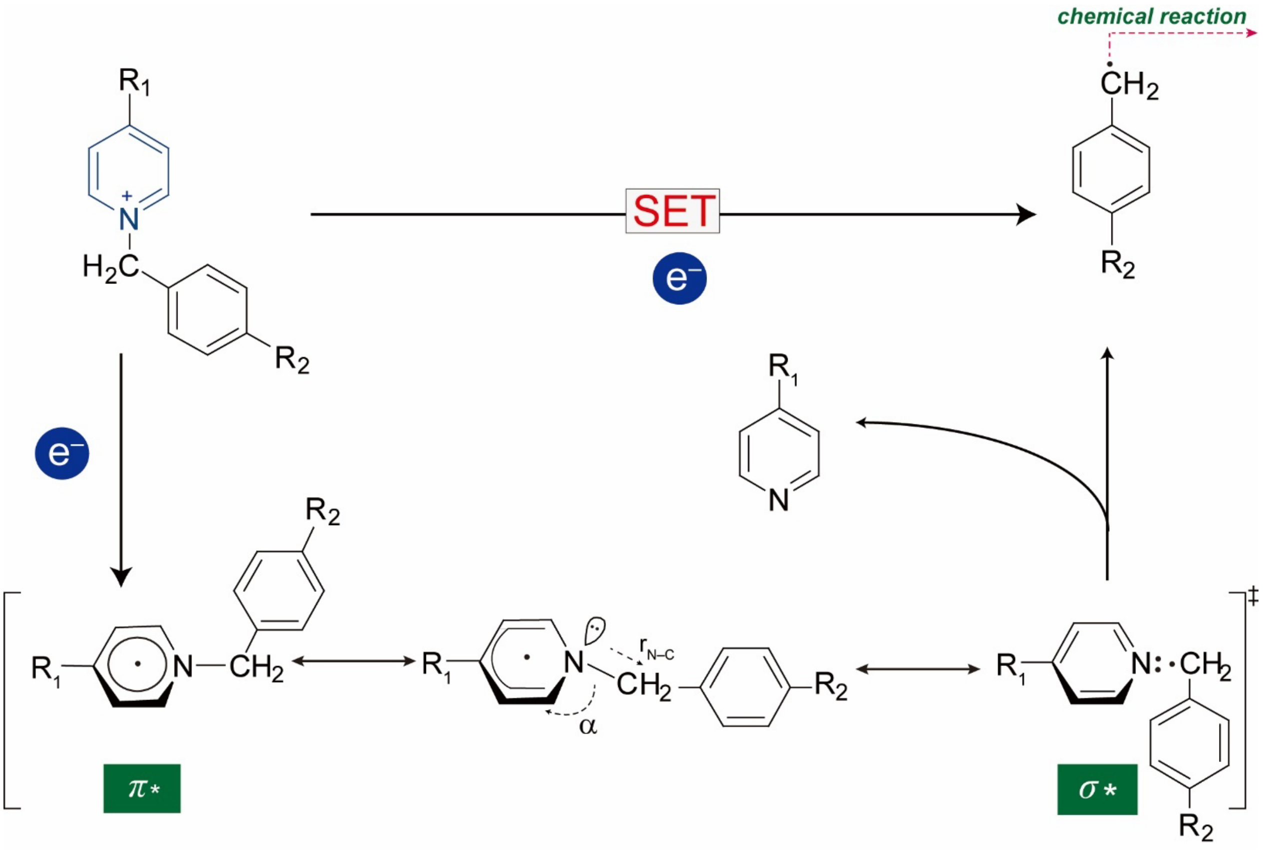

Supplementary Fig. 1 | A schematic diagram illustrating the mechanism of single electron transfer (SET) in Katritzky salts. SET enables the controlled release of synthetically important radicals and the generation of pyridine. On account of the orthogonality of the π* and σ* systems, direct bond elongation leads to an energetically unfavorable canonical intersection, resulting in high-energy excited-state products. According to previous research, the single-electron mechanism of an N-functionalized pyridinium salt introduces an additional electron into a π* orbital associated with the aromatic ring. Subsequently, the N–X bond elongates ($r_{N\text{-}X}$) and bends out of the plane (α), allowing the π* state to intersect with the dissociative σ* state. This intersection leads to the mixing of the pyridinyl π* and σ* orbitals, resulting in a three-electron N–X bond, which facilitates rapid fragmentation.

(1) General procedure A

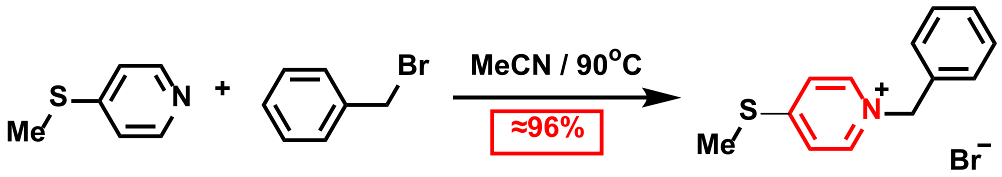

PyBz–1

Supplementary Fig. 2 | Synthesis of PyBz–1.

PyBz–1: To a solution of 4-(methylthio)pyridine (5.0 mmol, 1.0 eq.) in acetone (20 mL) was added benzyl bromide (5.5 mmol, 1.1 eq..) under nitrogen in a two-necked flask at 90 °C for 12 h, and then cooled to room temperature. Removal of the solvent under reduced pressure afforded the crude product, which was filtered, washed with dichloromethane (10 mL × 3), and dried under vacuum to afford the benzyl pyridinium salt PyBz–1 as a white solid in ≈96% yield. ^{1}H NMR (600 MHz, DMSO-d_6) δ 8.90 (d, J = 7.0 Hz, 2H), 7.96 (d, J = 7.1 Hz, 2H), 7.51 (d, J = 8.3 Hz, 2H), 7.47 – 7.37 (m, 3H), 5.72 (s, 2H), 2.70 (s, 3H). ^{13}C NMR (151 MHz, DMSO-d_6) δ 164.3, 142.7, 135.2, 129.6, 129.0, 123.4, 62.1, and 14.6. HRMS-ESI for PyBz–1; Calcd for $C_{13}H_{14}BrNS$: m/z = 216.0841 $[M-Br]^+$; Found 216.0842 $[M-Br]^+$.

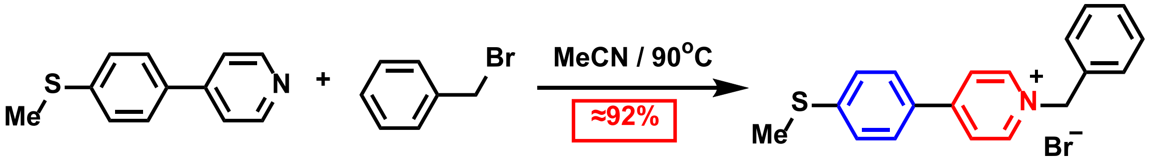

PyBz–2

Supplementary Fig. 3 | Synthesis of PyBz–2.

PyBz–2: This compound was prepared using the same methodology as used in the preparation of PyBz–1. PyBz–2 was isolated as a yellow solid with a yield in ≈92%. ^{1}H NMR (600 MHz, DMSO-d_6) δ 9.19 (d, J = 6.5 Hz, 2H), 8.52 (d, J = 6.4 Hz, 2H), 8.04 (d, J = 8.4 Hz, 2H), 7.57 (d, J = 6.8 Hz, 2H), 7.53 – 7.41 (m, 5H), 5.83 (s, 2H), 2.57 (s, 3H). ^{13}C NMR (151 MHz, DMSO-d_6) δ 154.8, 145.4, 145.0, 135.0, 129.8, 129.7, 129.6, 129.2, 129.0, 126.4, 124.5, 62.7, and 14.5. HRMS-ESI for PyBz–2; Calcd for $C_{19}H_{18}BrNS$: m/z =292.1154 $[M-Br]^+$; Found 292.1156 $[M-Br]^+$.

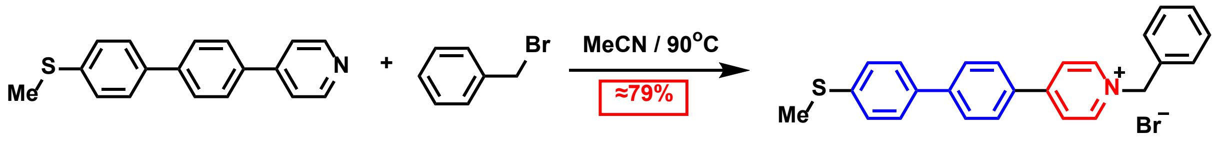

PyBz–3

Supplementary Fig. 4 | Synthesis of PyBz–3.

PyBz–3: This compound was prepared using the same methodology as used in the preparation of PyBz–1. PyBz–3 was isolated as a yellow solid with a yield in ≈79%. ^{1}H NMR (600 MHz, DMSO-d_6) δ 9.22 (d, J = 5.1 Hz, 2H), 8.59 (d, J = 6.4 Hz, 2H), 8.17 (s, 2H), 7.95 (d, J = 8.4 Hz, 2H), 7.78 (d, J = 8.4 Hz, 2H), 7.59 (d, J = 7.9 Hz, 2H), 7.43 (dd, J = 43.2, 7.9 Hz, 5H), 5.84 (s, 2H), 2.54 (s, 3H). ^{13}C NMR (151 MHz, DMSO-d_6) δ 154.9, 145.2, 143.5, 139.6, 135.2, 135.0, 132.5, 129.8, 129.7, 129.4, 129.2, 129.2, 127.8, 127.8, 126.8, 125.0, 62.8, and 15.0. HRMS-ESI for PyBz–3; Calcd for $C_{25}H_{22}BrNS$: m/z =368.1467 $[M - Br]^+$; Found 368.1468 $[M - Br]^+$.

(2) General procedure B

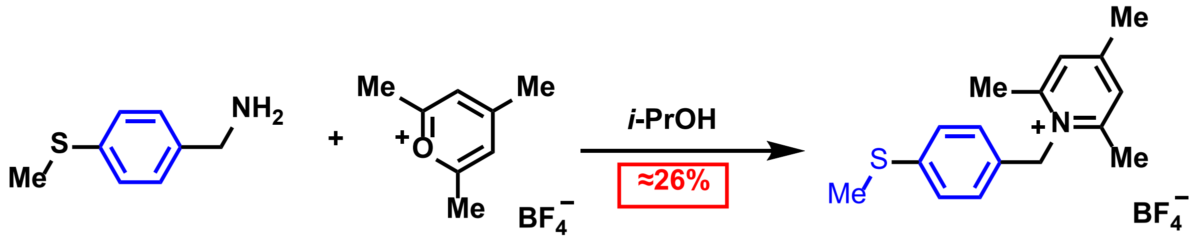

PhTMP–1

Supplementary Fig. 5 | Synthesis of PhTMP–1.

PhTMP–1: The (4-(methylthio)phenyl)methanamine (1.0 mmol, 1.0 eq.) was dissolved in anhydrous isopropyl alcohol (30 mL) by stirring. Subsequently, the corresponding 2,4,6-trimethylpyrylium salt (1.1 mmol, 1.1 eq..) was added. The mixture was then heated to 70 °C with stirring. After stirring for 8 hours, the solvent was evaporated under reduced pressure. The resulting residue was further purified through flash chromatography on a silica gel column using $MeOH/CH_2Cl_2$ (v/v = 1:10) as the eluent, yielding the product as a pale yellow oil with an

approximate yield of 26%. ^{1}H NMR (600 MHz, Chloroform-*d*) δ 7.53 (s, 2H), 7.22 (d, J = 8.1 Hz, 2H), 6.79 (d, J = 8.0 Hz, 2H), 5.74 (s, 2H), 2.72 (s, 6H), 2.58 (s, 3H), 2.46 (s, 3H). ^{13}C NMR (151 MHz, DMSO-d_6) δ 158.7, 155.3, 139.0, 129.5, 128.9, 127.0, 126.7, 54.7, 21.5, 21.2, and 15.0. HRMS-ESI for PhTMP–1; Calcd for $C_{16}H_{20}BrNS$: m/z =258.1311 $[M - BF_4^-]^+$; Found 258.1313 $[M - BF_4^-]^+$.

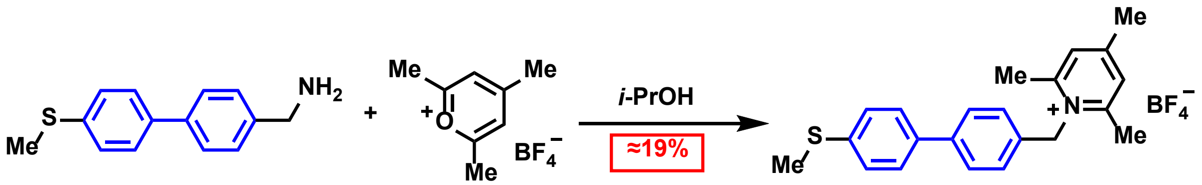

PhTMP–2

Supplementary Fig. 6 | Synthesis of PhTMP–2.

PhTMP–2: This compound was prepared using the same methodology as used in the preparation of PhTMP–1. PhTMP–2 was isolated as a yellow solid with a yield in ≈19%.^{1}H NMR (600 MHz, Chloroform-*d*) δ 7.56 (d, J = 8.3 Hz, 2H), 7.49 (s, 2H), 7.46 (d, J = 8.0 Hz, 2H), 7.32 (d, J = 8.2 Hz, 2H), 6.92 (d, J = 7.9 Hz, 2H), 5.83 (s, 2H), 2.77 (s, 6H), 2.60 (s, 3H), 2.52 (s, 3H). ^{13}C NMR (151 MHz, Chloroform-*d*) δ 158.8, 155.2, 141.0, 138.6, 136.4, 130.4, 128.8, 128.0, 127.3, 126.8, 125.6, 55.2, 21.7, 21.2, and 15.7. HRMS-ESI for PhTMP–2; Calcd for $C_{22}H_{24}BrNS$: m/z =334.1624 $[M - BF_4^-]^+$; Found 334.1626 $[M - BF_4^-]^+$.

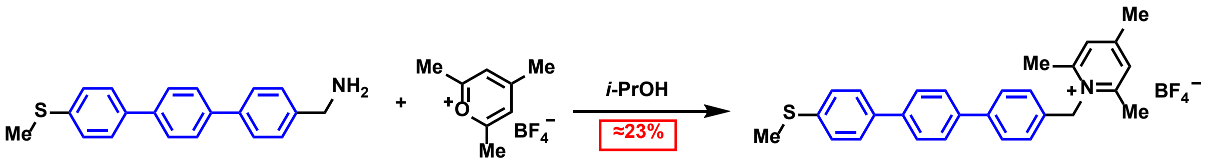

PhTMP–3

Supplementary Fig. 7 | Synthesis of PhTMP–3.

PhTMP–3: This compound was prepared using the same methodology as used in the preparation of PhTMP–1. PhTMP–3 was isolated as a yellow solid with a yield in ≈23%.^{1}H NMR (600 MHz,

Chloroform-*d*) δ 7.69 – 7.59 (m, 6H), 7.56 (d, J = 7.8 Hz, 3H), 7.52 (s, 2H), 7.34 (d, J = 8.1 Hz, 2H), 6.97 (d, J = 8.1 Hz, 2H), 5.88 (s, 2H), 2.80 (s, 6H), 2.62 (s, 3H), 2.53 (s, 3H). ^{13}C NMR (151 MHz, Chloroform-*d*) δ 158.5, 155.4, 141.3, 140.1, 138.5, 138.1, 137.1, 130.6, 128.8, 128.6, 128.2, 127.4, 127.3, 127.0, 125.6, 55.4, 21.8, 21.4, and 15.8. HRMS-ESI for PhTMP–3; Calcd for $C_{28}H_{28}BrNS$: *m/z* =410.1937 [$M - BF_4^-$]$^+$; Found 410.1940 [$M - BF_4^-$]$^+$.

(3) General procedure C

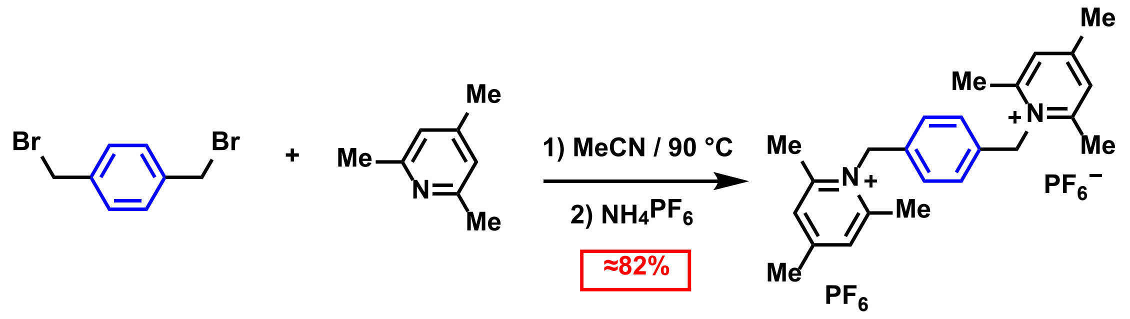

Ph(TMP)2–1

Supplementary Fig. 8 | Synthesis of Ph(TMP)2–1.

Ph(TMP)2–1: To a solution of 1,4-bis(bromomethyl)benzene (2.0 mmol, 1.0 eq.) in MeCN (20 mL) was added 2,4,6-trimethylpyridine (in excess) in a flask at 90 °C for 12 h. After consumption of the starting material, the reaction mixture was allowed to cool to room temperature. Removal of the solvent under reduced pressure afforded the crude product, which was filtered, washed with diethyl ether and dried under reduced pressure to afford the benzyl pyridinium salt as a white solid. The solid was dissolved in water and ammonium hexafluorophosphate was then added to the solution. The precipitated pyridinium salt was collected by filtration, washed, and dried to obtain the Ph(TMP)2–1 with a yield in ≈82%. ^{1}H NMR (600 MHz, Acetonitrile-d_3) δ 7.63 (s, 4H), 6.98 (s, 4H), 5.67 (s, 4H), 2.61 (s, 12H), 2.55 (s, 6H); ^{13}C NMR (151 MHz, Acetonitrile-d_3) δ 160.1, 155.7, 133.2, 129.5, 127.2, 55.3, 21.4, 21.2. HRMS-ESI for Ph(TMP)2–1; Calcd for $C_{24}H_{30}F_{12}N_2P_2$: *m/z* =491.2045 [$M - PF_6^-$]$^+$; Found 491.2045 [$M - PF_6^-$]$^+$.

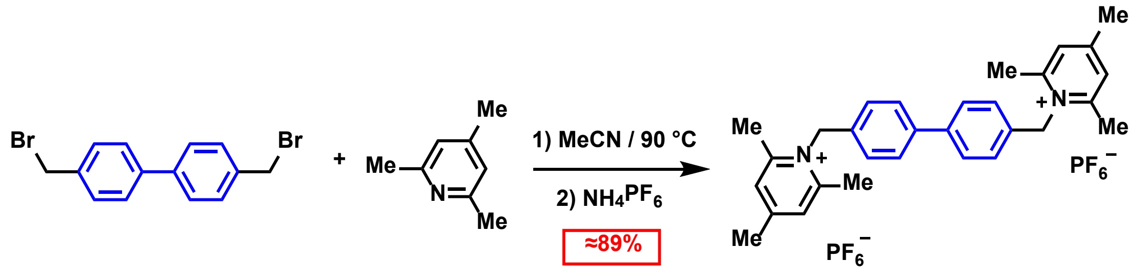

Ph(TMP)2–2

Supplementary Fig. 9 | Synthesis of Ph(TMP)$_2$–2.

Ph(TMP)$_2$–2: This compound was prepared using the same methodology as used in the preparation of Ph(TMP)$_2$-1. Ph(TMP)$_2$-2 was isolated as a yellow solid with a yield in ≈89%. ^{1}H NMR (600 MHz, Acetonitrile-*d*$_3$) δ 7.68 – 7.65 (m, 8H), 7.06 (d, J = 8.1 Hz, 4H), 5.73 (s, 4H), 2.66 (s, 12H), 2.57 (s, 6H). ^{13}C NMR (151 MHz, Acetonitrile- *d*$_3$) δ 160.0, 155.8, 140.6, 132.2, 129.5, 128.5, 126.8, 55.5, 21.4, 21.3. HRMS-ESI for Ph(TMP)$_2$–2; Calcd for $C_{24}H_{30}F_{12}N_2P_2$: *m/z* =567.2358 [*M* – PF6$^-$]+; Found 567.2365 [*M* – PF6$^-$]+.

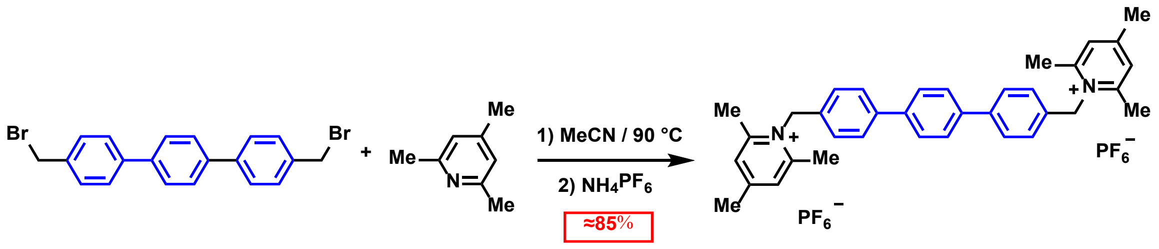

Ph(TMP)3-3

Supplementary Fig. 10 | Synthesis of Ph(TMP)$_3$-3.

Ph(TMP)$_3$-3: This compound was prepared using the same methodology as used in the preparation of Ph(TMP)$_2$-1. Ph(TMP)$_3$-2 was isolated as a yellow solid with a yield in ≈85%. ^{1}H NMR (600 MHz, Acetonitrile-*d*$_3$) δ 7.78 – 7.72 (m, 8H), 7.66 (s, 4H), 7.07 (d, *J* = 8.0 Hz, 4H), 5.74 (s, 4H), 2.68 (s, 12H), 2.57 (s, 6H). ^{13}C NMR (151 MHz, Acetonitrile-*d*$_3$) δ 160.0, 155.7, 140.9, 139.7, 132.0, 129.4, 128.3, 128.1, 126.7, 55.4, 21.4, 21.2. HRMS-ESI for Ph(TMP)$_3$-3; Calcd for $C_{36}H_{38}F_{12}N_2P_2$: *m/z* =643.2671 [*M* – PF$_6^-$]$^+$; Found 643.2681 [*M* – PF$_6^-$]$^+$.

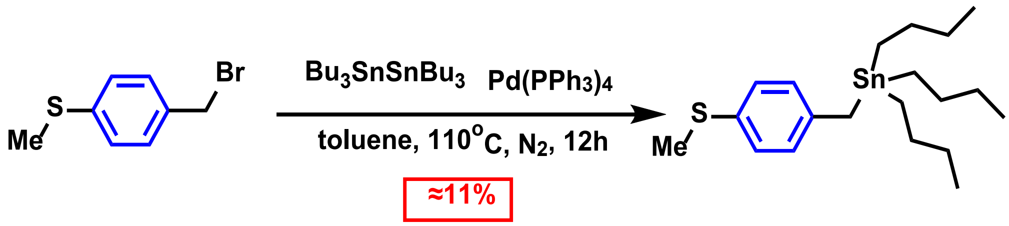

PhSn–1

Supplementary Fig. 11 | Synthesis of PhSn–1.

PhSn–1: A 100 mL round-bottom flask, equipped with a magnetic stirrer, was charged with **(4-**(bromomethyl)phenyl)(methyl)sulfane (2.6 mmol, 1.0 eq.), $Bu_3SnSnBu_3$ (3.1 mmol, 1.2 eq.), $Pd(PPh_3)_4$(0.26 mmol, 0.1 eq.), toluene (60 mL). The reaction mixture was stirred at 110 °C under an N_2 atmosphere for 12 h, cooled to room temperature, and passed through a pad of Celite. Then, 60 mL of water were added to the mixture and the solution extracted with DCM (60 mL × 3). The combined organic layers were washed with brine, dried over Na_2SO_4 and evaporated in vacuo. The product was purified by silica gel chromatography using hexane as the eluent to give PhSn–1 as a transparent liquid with a yield in ≈11%.^{1}H NMR (600 MHz, Chloroform-*d*) δ 7.12 (d, J = 8.2 Hz, 2H), 6.91 (d, J = 7.9 Hz, 2H), 2.44 (s, 3H), 2.26 (s, 2H), 1.46 – 1.35 (m, 6H), 1.25 (h, J = 7.2 Hz, 6H), 0.86 (t, J = 7.3 Hz, 9H), 0.83 – 0.74 (m, 6H).^{13}C NMR (151 MHz, Chloroform-*d*) δ 141.5, 131.4, 128.2, 127.6, 29.0, 27.3, 17.8, 17.3, 13.7, 9.4. HRMS (MALDI-TOF) for PhSn–1; Calcd for $C_{20}H_{36}SSn$: *m/z* =428.1560; Found 428.1542.

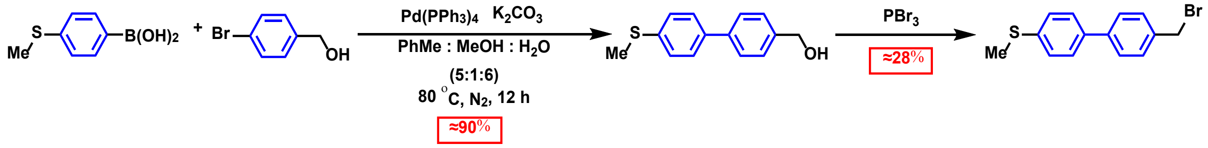

PhOH–2 PhBr–2

Supplementary Fig. 12 | Synthesis of PhBr–2.

PhOH–2: A 250 mL round-bottom flask, equipped with a magnetic stirrer, was charged with (4-(methylthio)phenyl)boronic acid (15 mmol, 1.0 eq.), (4-bromophenyl)methanol (18 mmol, 1.2 eq.)

$Pd(PPh_3)_4$ (0.15 mmol, 0.01 eq.), K_2CO_3 (45 mmol, 3.0 eq.), toluene (75 mL), MeOH (15 mL) and H_2O (90 mL). The reaction mixture was stirred at 80 °C under an N_2 atmosphere for 12 h, cooled to room temperature, and passed through a pad of Celite. Then, 60 mL of water were added to the mixture and the solution extracted with ethyl acetate (60 mL × 3). The combined organic layers were washed with brine, dried over Na_2SO_4 and evaporated in vacuo to give the crude product PhOH–2 as a white solid which was used for the next reaction without purification.

PhBr–2: To a solution of PhOH–2 (8.6 mmol, 1.0 eq.) in dichloromethane (20 mL, 0.05 M) cooled at 0°C, PBr_3 (25.8 mmol, 3.0 eq.) was added. The mixture was stirred for 5 h at 0°C and then slowly allowed to warm up to RT over the following 10 h, cooled to room temperature. Then, 60 mL of ice water were added to the mixture and the solution extracted with dichloromethane (60 mL × 3). The combined organic layers were washed with brine, dried over Na_2SO_4 and evaporated in vacuo. The product was purified by silica gel chromatography using petroleum ether/dichloromethane (10:1) as the eluent to give PhBr–2 as a white solid with a yield of 28%. ^{1}H NMR (600 MHz, Chloroform-*d*) δ 7.55 (d, J = 7.8 Hz, 2H), 7.51 (d, J = 7.9 Hz, 2H), 7.46 (d, J = 7.8 Hz, 2H), 7.32 (d, J = 8.1 Hz, 2H), 4.55 (s, 2H), 2.52 (s, 3H).

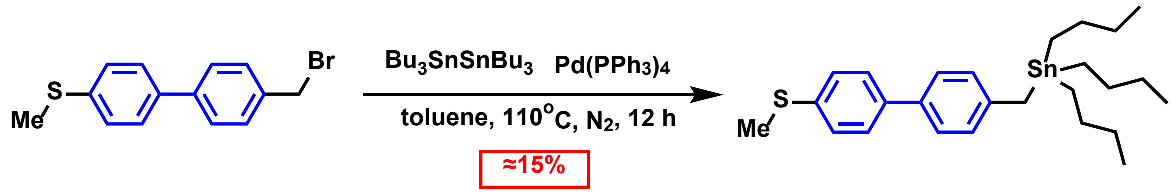

PhSn–2

Supplementary Fig. 13 | Synthesis of PhSn-2.

PhSn–2: This compound was prepared using the same methodology as used in the preparation of PhSn–1. PhSn–2 was isolated as a yellow solid with a yield in ≈15%. ^{1}H NMR (600 MHz, Chloroform-*d*) δ 7.49 (d, J = 8.3 Hz, 2H), 7.39 (d, J = 8.1 Hz, 2H), 7.30 (d, J = 8.4 Hz, 2H), 7.04 (d, J = 8.1 Hz, 2H), 2.51 (s, 3H), 2.34 (s, 2H), 1.47 – 1.36 (m, 6H), 1.26 (t, J = 7.4 Hz, 6H), 0.89 –

0.80 (m, 15H). ^{13}C NMR (151 MHz, Chloroform-*d*) δ 143.1, 138.4, 136.5, 135.0, 127.4, 127.2, 127.0, 126.6, 29.0, 27.3, 18.0, 16.2, 13.7, 9.4. HRMS (MALDI-TOF); calculated for PhSn–2**:** [M-Bu]$^+$ 448.1247, found 448.1244.

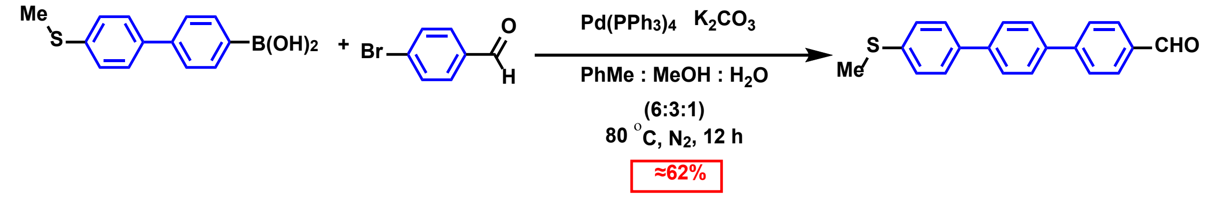

PhCHO-3

Supplementary Fig. 14 | Synthesis of PhCHO-3.

PhCHO–3: A 250 mL round-bottom flask, equipped with a magnetic stirrer, was charged with 4-bromobenzaldehyde (4.8 mmol, 1.0 eq.), (4'-(methylthio)-[1,1'-biphenyl]-4-yl)boronic acid (5.7 mmol, 1.2 eq..), $Pd(PPh_3)_4$ (0.04 mmol, 0.01 eq.), K_2CO_3 (14.2 mmol, 3.0 eq.), toluene (25 mL), MeOH (5 mL) and H_2O (30 mL). The reaction mixture was stirred at 80 °C under an N_2 atmosphere for 12 h, cooled to room temperature, and passed through a pad of Celite. Then, 20 mL of water were added to the mixture and the solution extracted with ethyl acetate (20 mL × 3). The combined organic layers were washed with brine, dried over Na_2SO_4 and evaporated in vacuo to give PhCHO–3 as a white solid with a yield of 62%. ^1H NMR (600 MHz, Chloroform-*d*) δ 10.07 (s, 1H), 7.98 (d, J = 6.7 Hz, 2H), 7.81 (d, J = 7.6 Hz, 2H), 7.71 (m, J = 8.5, 1.6 Hz, 4H), 7.58 (d, J = 6.8 Hz, 2H), 7.36 (d, J = 6.9 Hz, 2H), 2.54 (s, 4H).

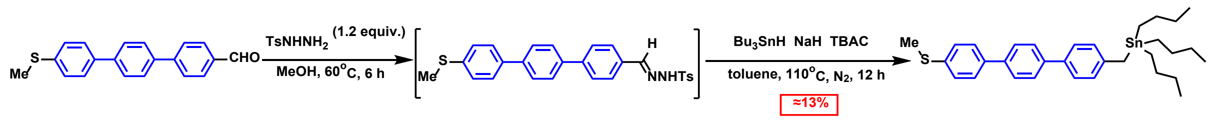

PhSn–3

Supplementary Fig. 15 | Synthesis of PhSn-3.

N-Tosylhydrazone: A 200 mL round-bottom flask, equipped with a magnetic stirrer, was charged with PhCHO-3 (2.0 mmol, 1.0 eq..), $TsNHNH_2$ (2.2 mmol, 1.1 eq.) and MeOH (20 mL). The solution was sealed and heated at 60 °C for 6 h. After the reaction was cooled to room temperature. Removal of the solvent under reduced pressure and washed with ice MeOH afforded the N-Tosylhydrazone product which was used for the next reaction without purification.

PhSn–3: A 150 mL round-bottom flask, equipped with a magnetic stirrer, was charged with N-tosylhydrazone of PhCHO-3 (1.0 mmol, 1.0 eq.), NaH (1.1 mmol, 1.1 eq.), and TBAC (0.2 mmol, 0.2 eq.) were weighed in a 10 mL Schlenk tube. Toluene (3 mL) was then added. The solution was stirred at room temperature for 10 min. Then $^{n}Bu_3SnH$ (1.0 mmol, 1,0 eq.) was added to the reaction system through a syringe. The resulting reaction solution was stirred at 110 °C for 12 h. The reaction mixture was then filtered through silica gel with petroleum ether as eluent. The combined filtrate was concentrated on a rotary evaporator under reduced pressure to leave a crude residue, which was purified by flash chromatography with silica gel (eluted with petroleum ether). PhSn–3 was obtained as colorless liquid with a yield of 13%.^{1}H NMR (600 MHz, Chloroform-d) δ 7.65 (t, J = 5.8 Hz, 4H), 7.57 (d, J = 8.0 Hz, 2H), 7.54 (d, J = 7.6 Hz, 2H), 7.34 (d, J = 8.0 Hz, 2H), 7.27 (s, 2H), 2.53 (s, 3H), 2.41 (s, 2H), 1.30 – 1.28 (m, 6H), 1.26 (s, 6H), 0.89 – 0.86 (m, 9H), 0.84 (s, 6H).^{13}C NMR (151 MHz, Chloroform-*d*) δ 140.0, 139.1, 137.8, 137.6, 137.6, 137.2, 129.5, 127.3, 127.1, 127.0, 126.9, 31.9, 29.7, 22.7, 21.1, 15.9, 14.1. HRMS (MALDI-TOF) for PhSn–3; Calcd for $C_{32}H_{44}SSn$: m/z =580.2186; Found 580.2185.

Supplementary Note 2. NMR spectroscopy

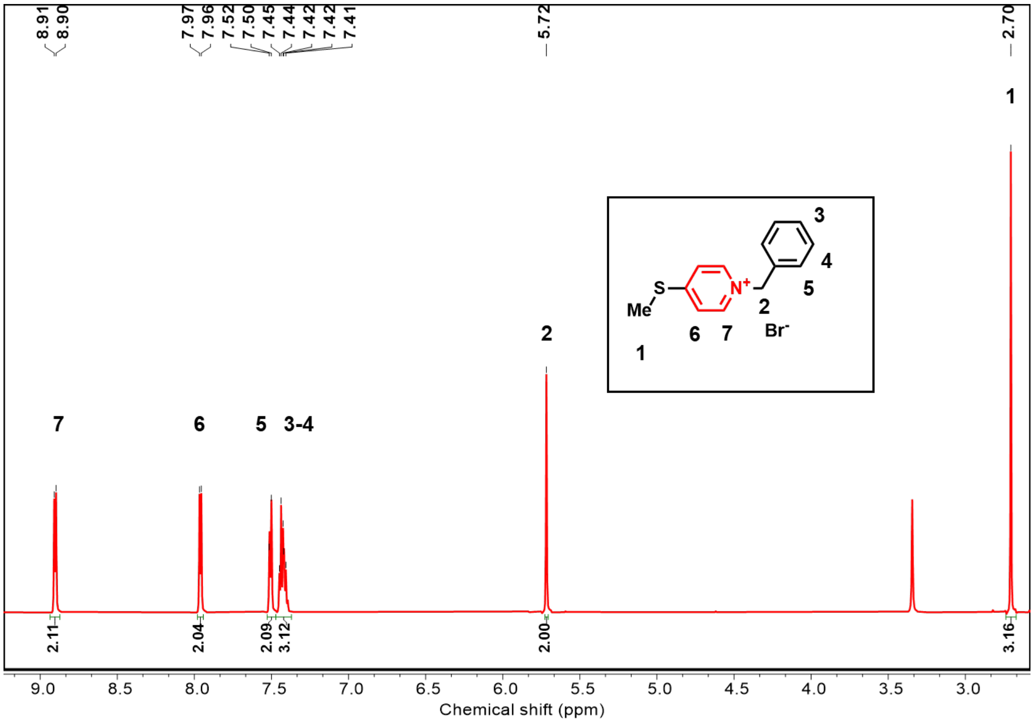

Supplementary Fig. 16 | ^{1}H NMR (600 MHz, DMSO-d_6, 298 K) of PyBz–1.

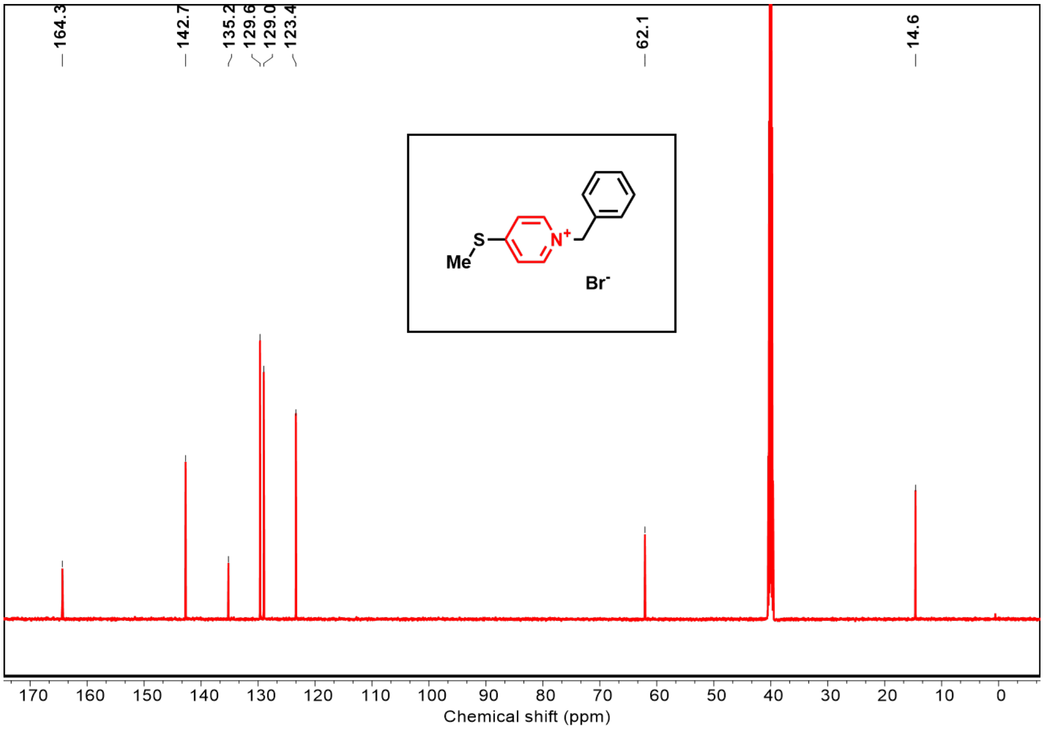

Supplementary Fig. 17 | ^{13}C NMR spectrum (151 MHz, DMSO-d_6, 298 K) of PyBz–1.

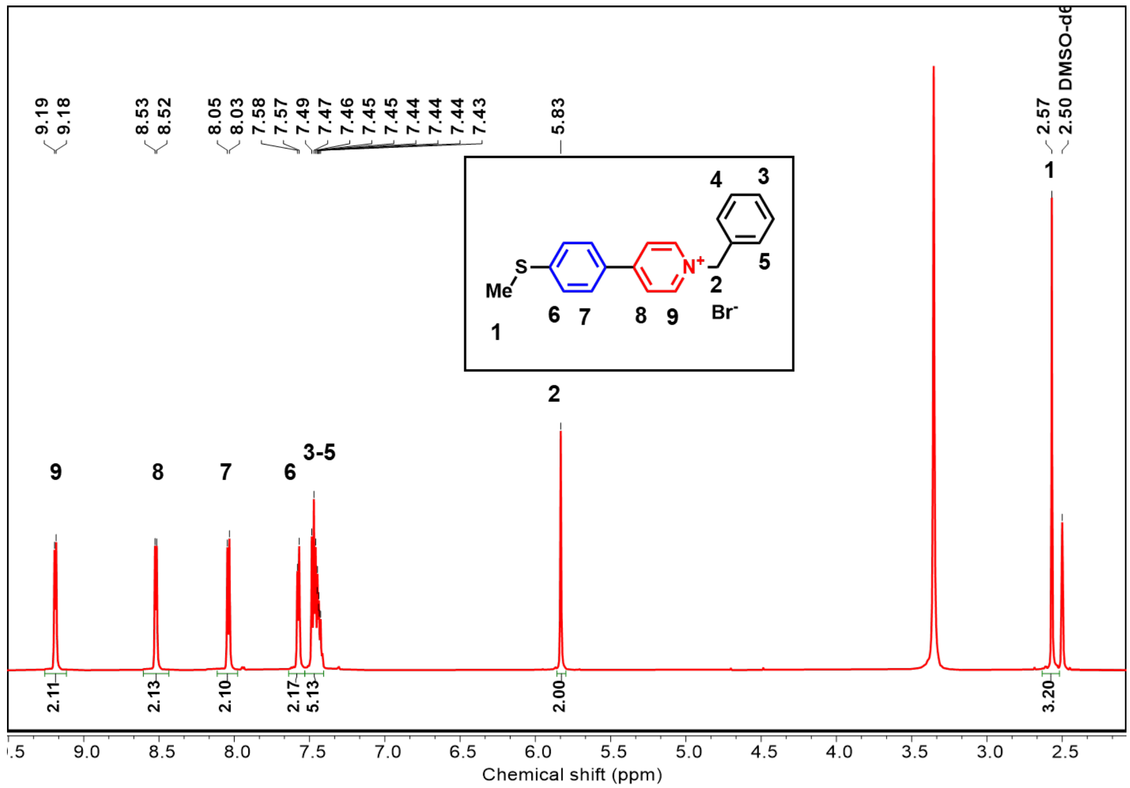

Supplementary Fig. 18 | ^{1}H NMR (600 MHz, DMSO-d_6, 298 K) of PyBz–2.

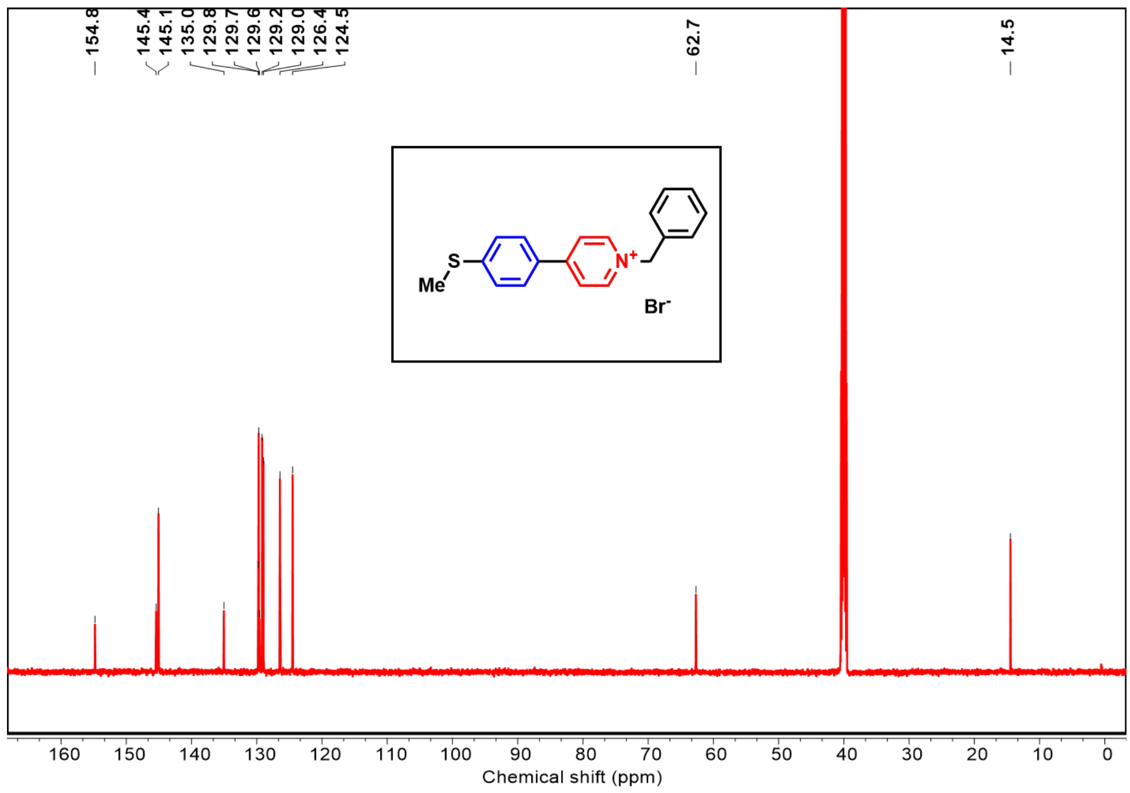

Supplementary Fig. 19 | ^{13}C NMR spectrum (151 MHz, DMSO-d_6, 298 K) of PyBz–2.

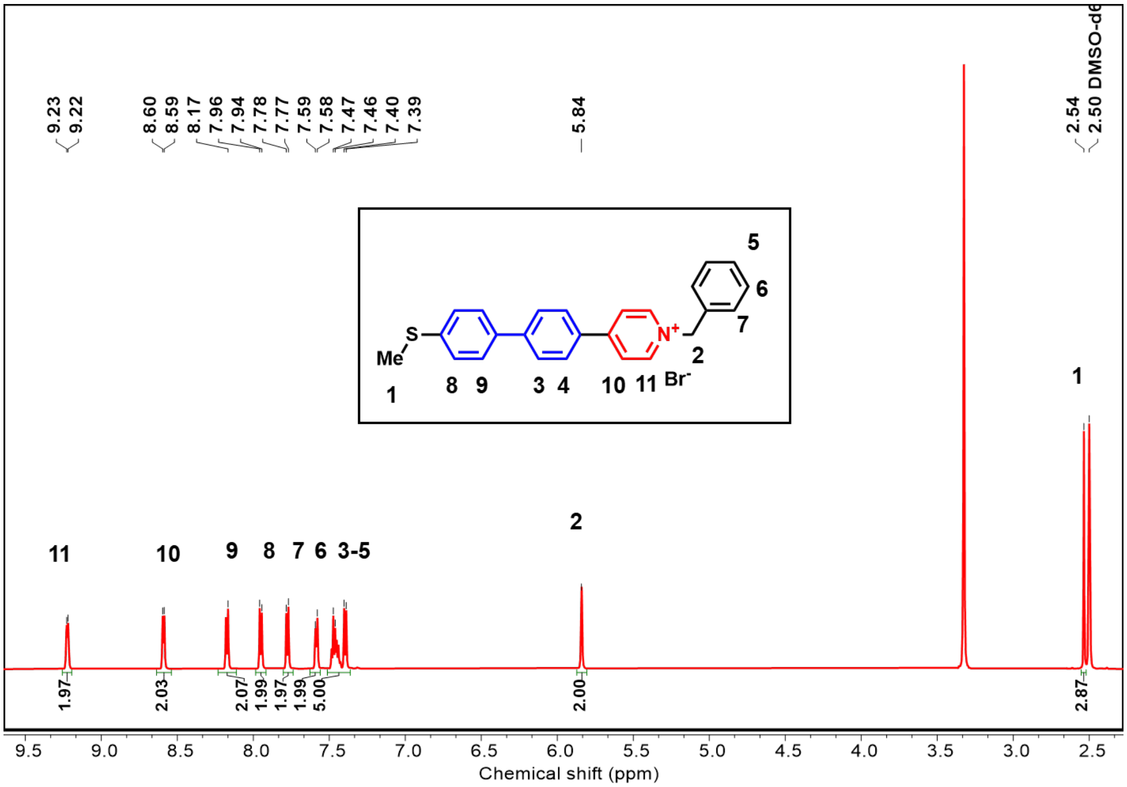

Supplementary Fig. 20 | ^{1}H NMR (600 MHz, DMSO-*d*$_6$, 298 K) of PyBz–3.

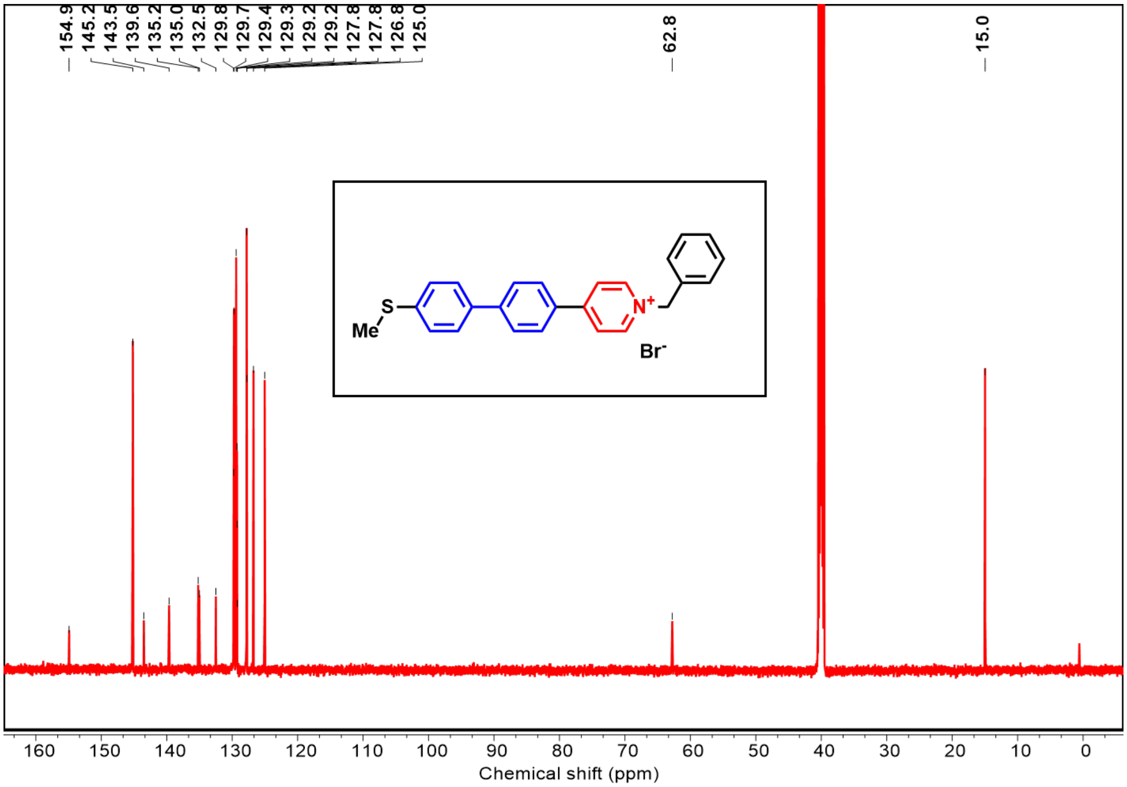

Supplementary Fig. 21 | ^{13}C NMR spectrum (151 MHz, DMSO-*d*$_6$, 298 K) of PyBz–3.

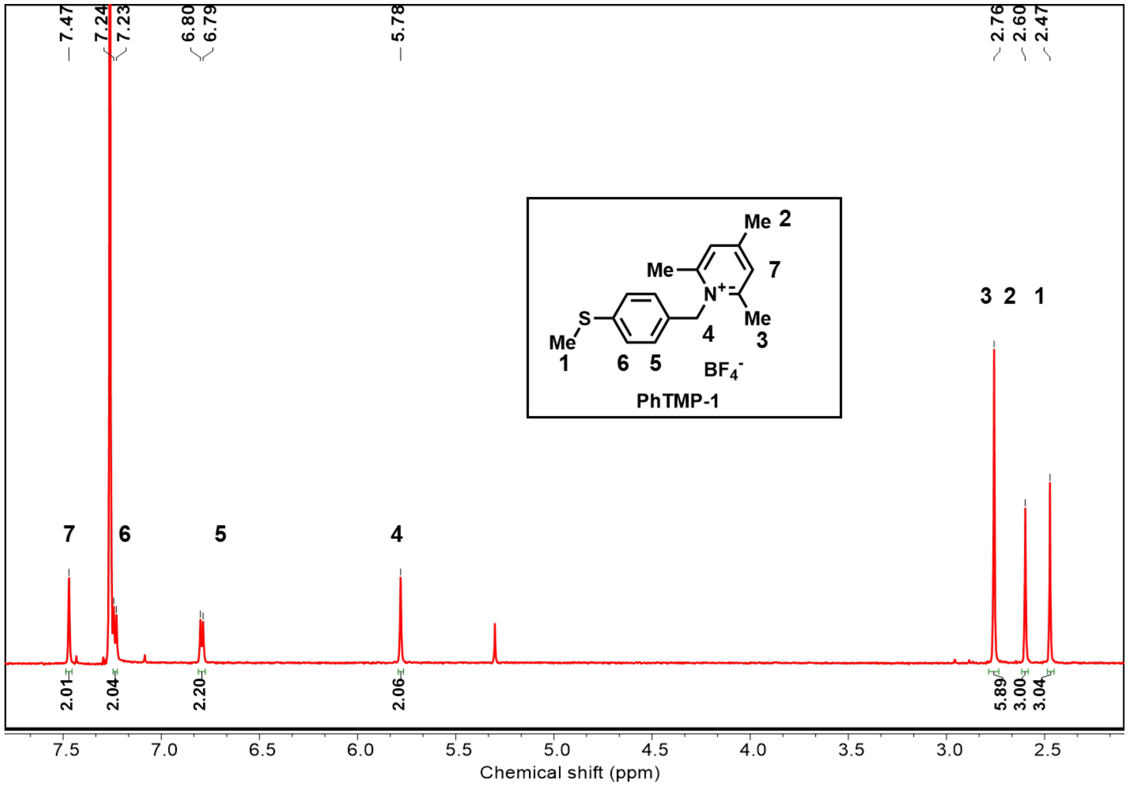

Supplementary Fig. 22 | ^{1}H NMR (600 MHz, Chloroform-*d*, 298 K) of PhTMP–1.

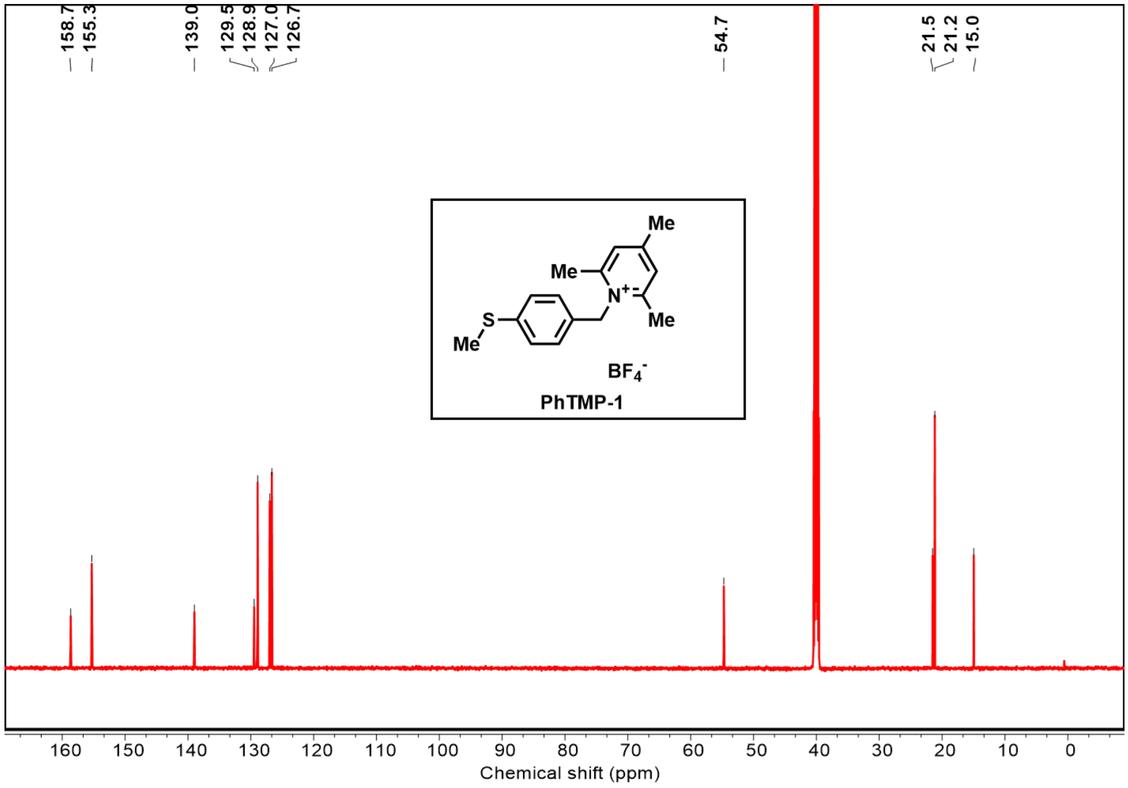

Supplementary Fig. 23 | ^{13}C NMR spectrum (151 MHz, DMSO-d_6, 298 K) of PhTMP–1.

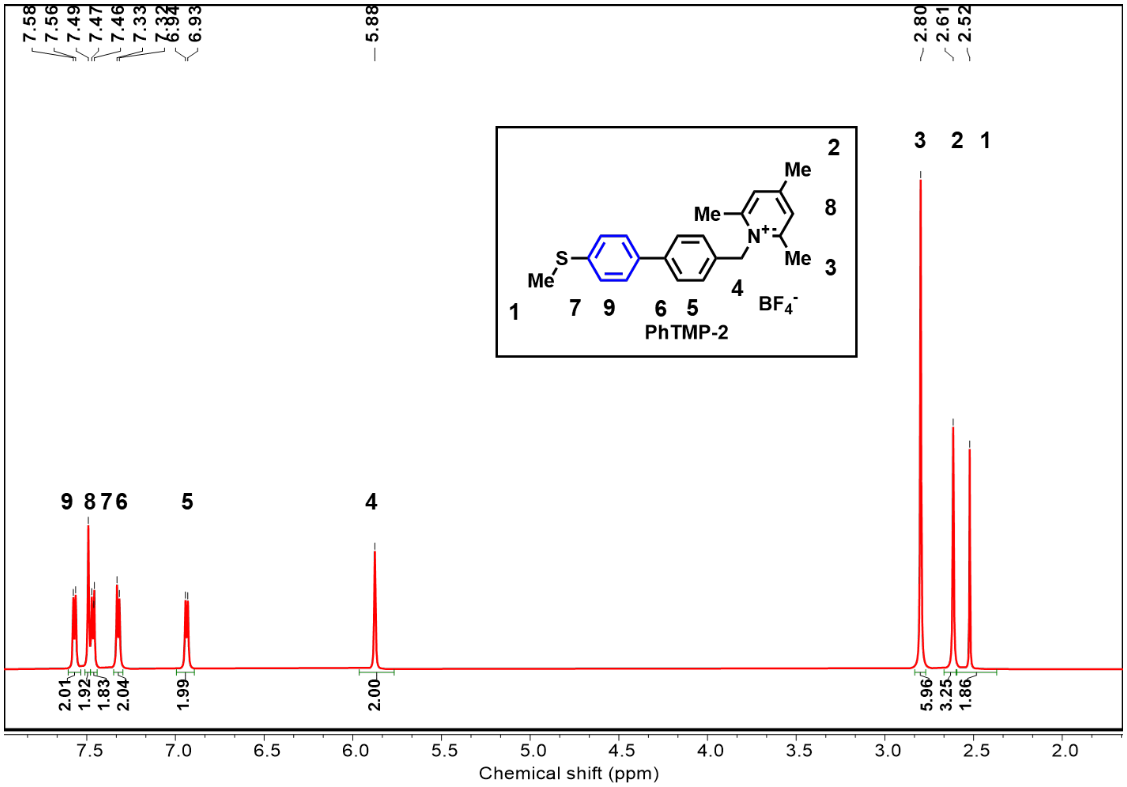

Supplementary Fig. 24 | ^{1}H NMR (600 MHz, Chloroform-*d*, 298 K) of PhTMP–2.

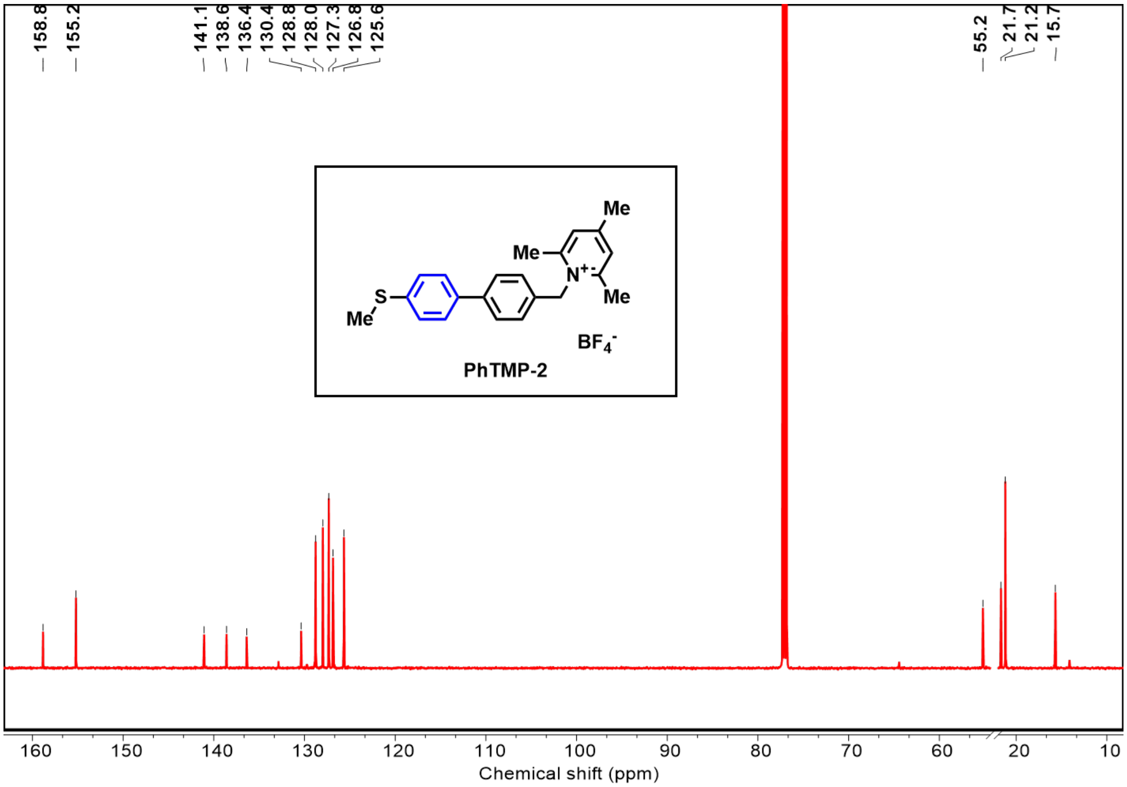

Supplementary Fig. 25 | ^{13}C NMR spectrum (151 MHz, Chloroform-*d*, 298 K) of PhTMP–2.

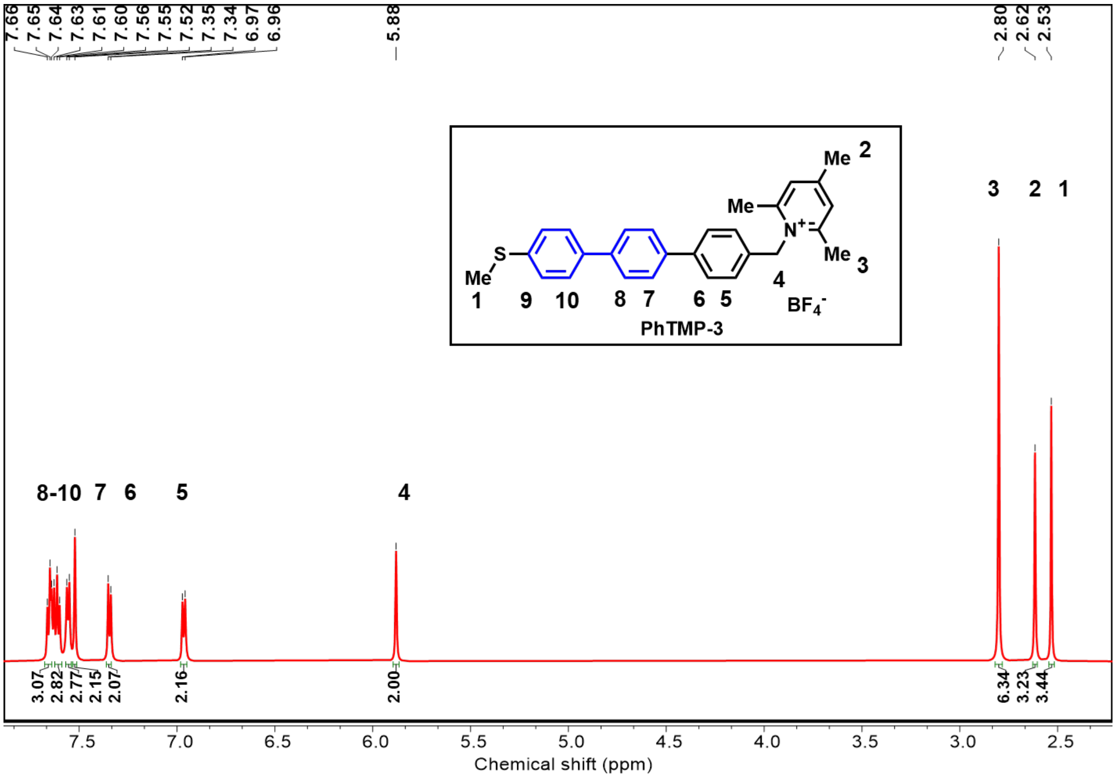

Supplementary Fig. 26 | ^{1}H NMR (600 MHz, Chloroform-*d*, 298 K) of PhTMP–3.

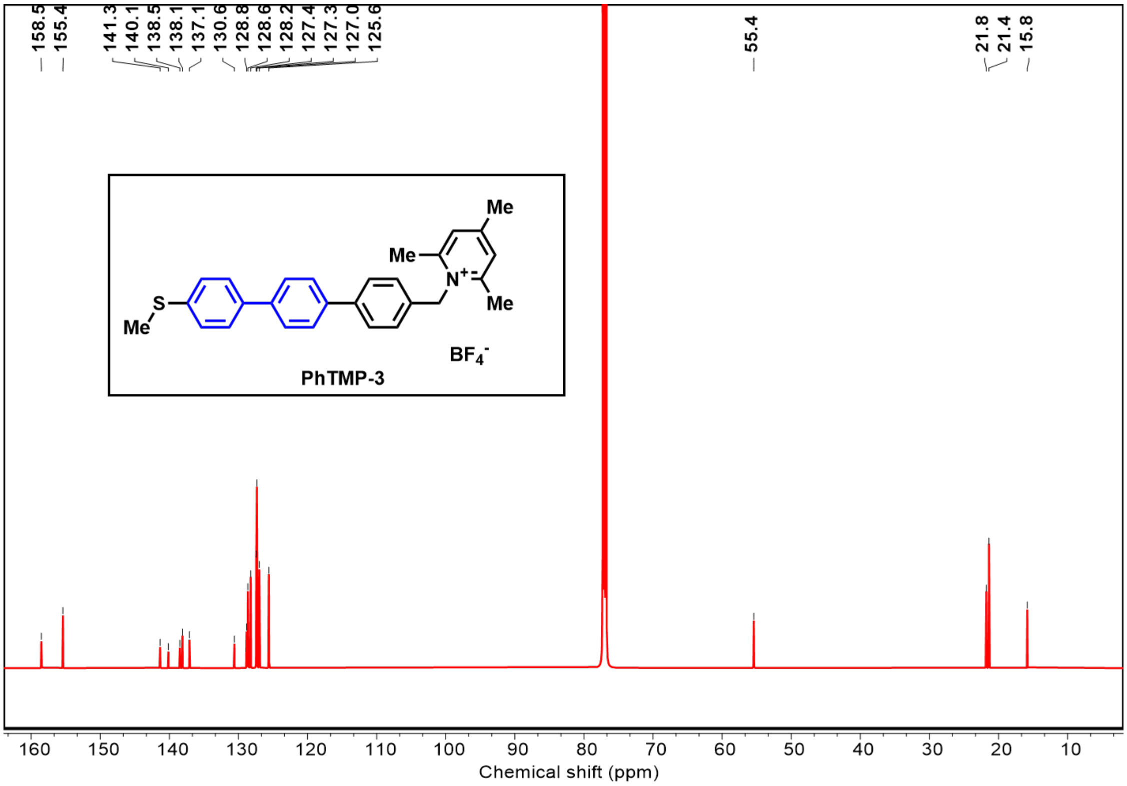

Supplementary Fig. 27 | ^{13}C NMR spectrum (151 MHz, Chloroform-*d*, 298 K) of PhTMP–3.

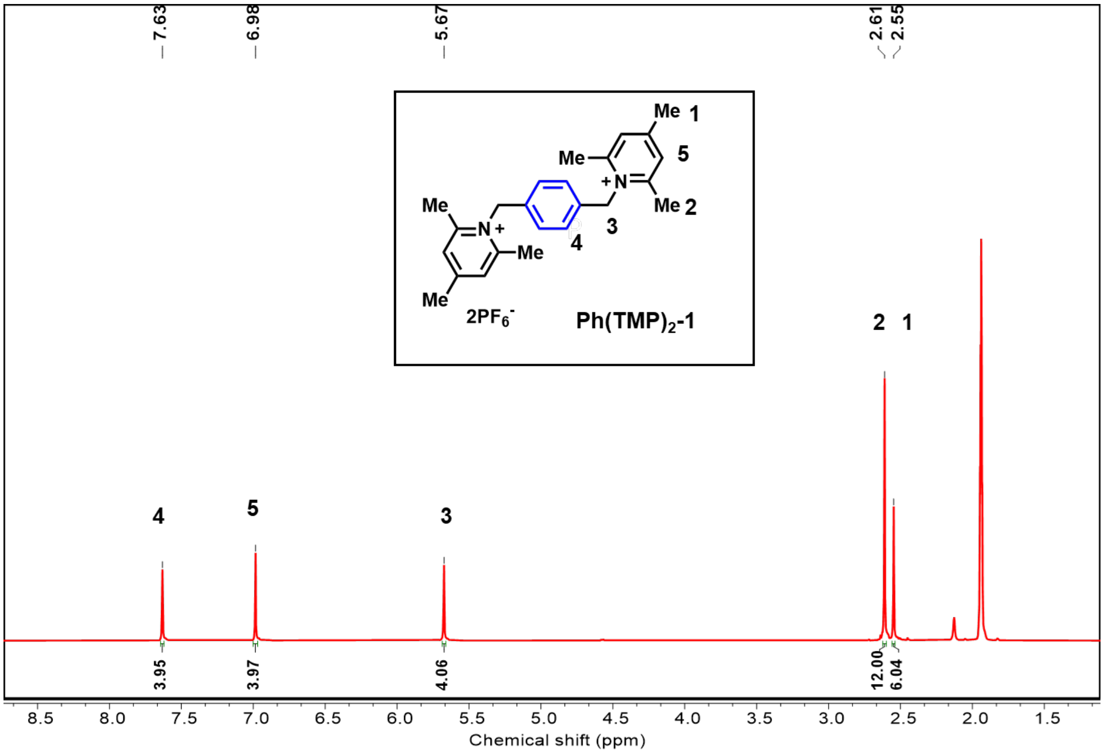

Supplementary Fig. 28 | ^{1}H NMR (600 MHz, Acetonitrile- d_3, 298 K) of Ph(TMP)$_2$–1.

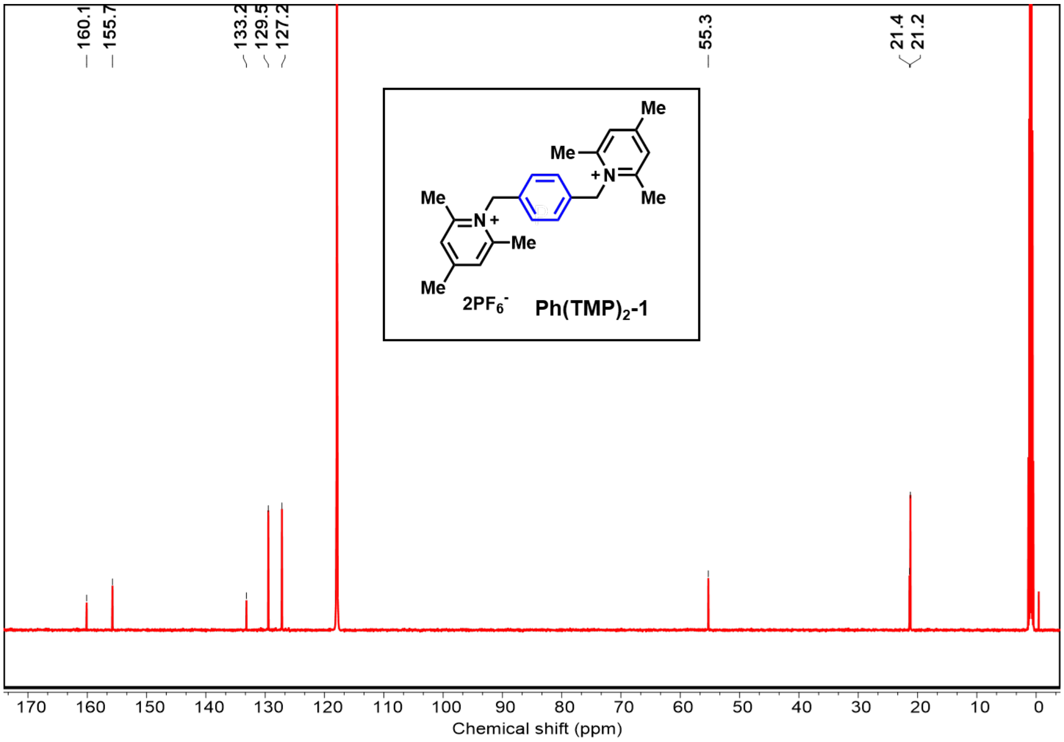

Supplementary Fig. 29 |^{13}C NMR spectrum (151 MHz, Acetonitrile-d_3, 298 K) of Ph(TMP)$_2$–1.

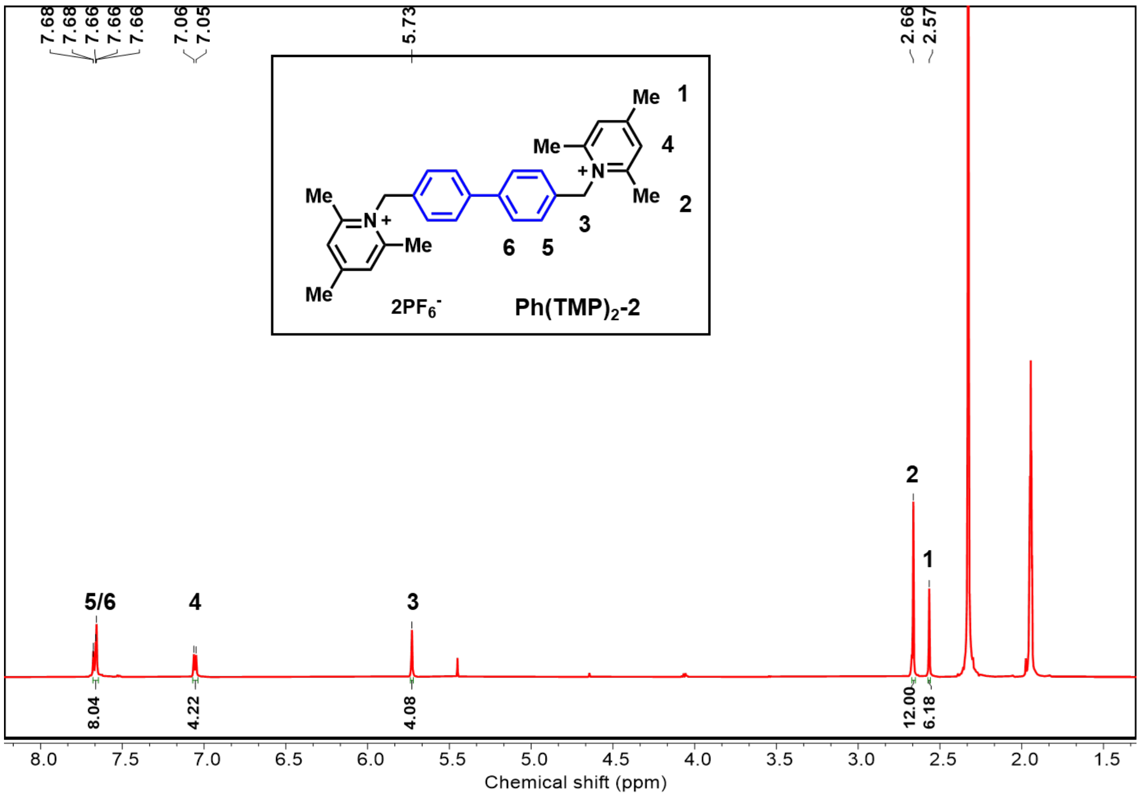

Supplementary Fig. 30 | ^{1}H NMR (600 MHz, Acetonitrile- d_3, 298 K) of Ph(TMP)$_2$–2.

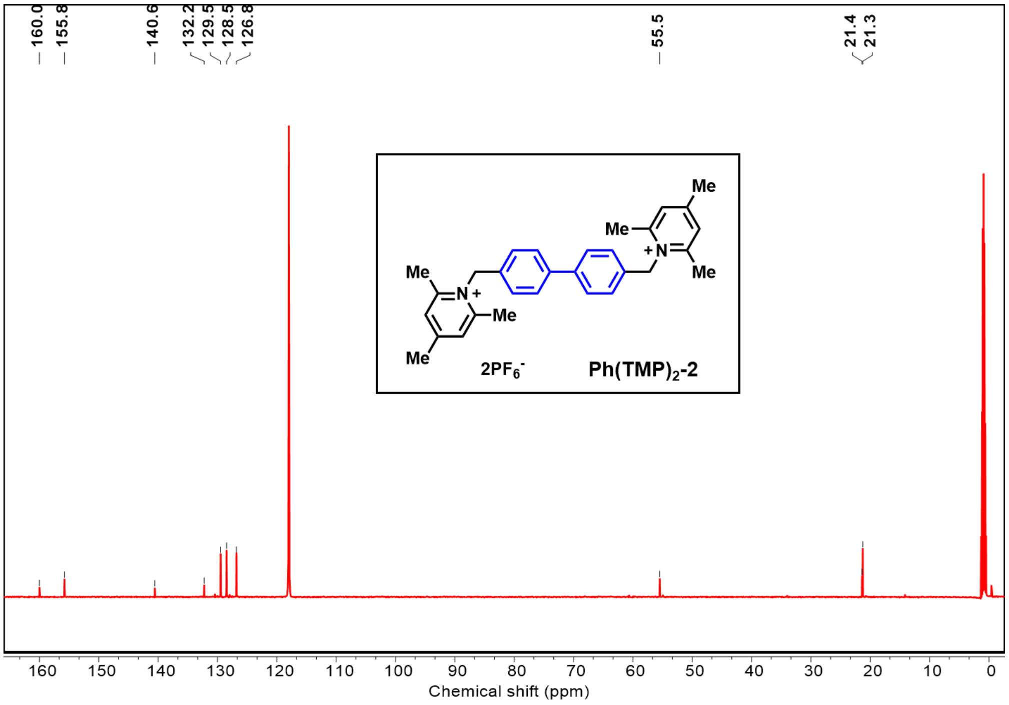

Supplementary Fig. 31 |^{13}C NMR spectrum (151 MHz, Acetonitrile-d_3, 298 K) of Ph(TMP)$_2$–2.

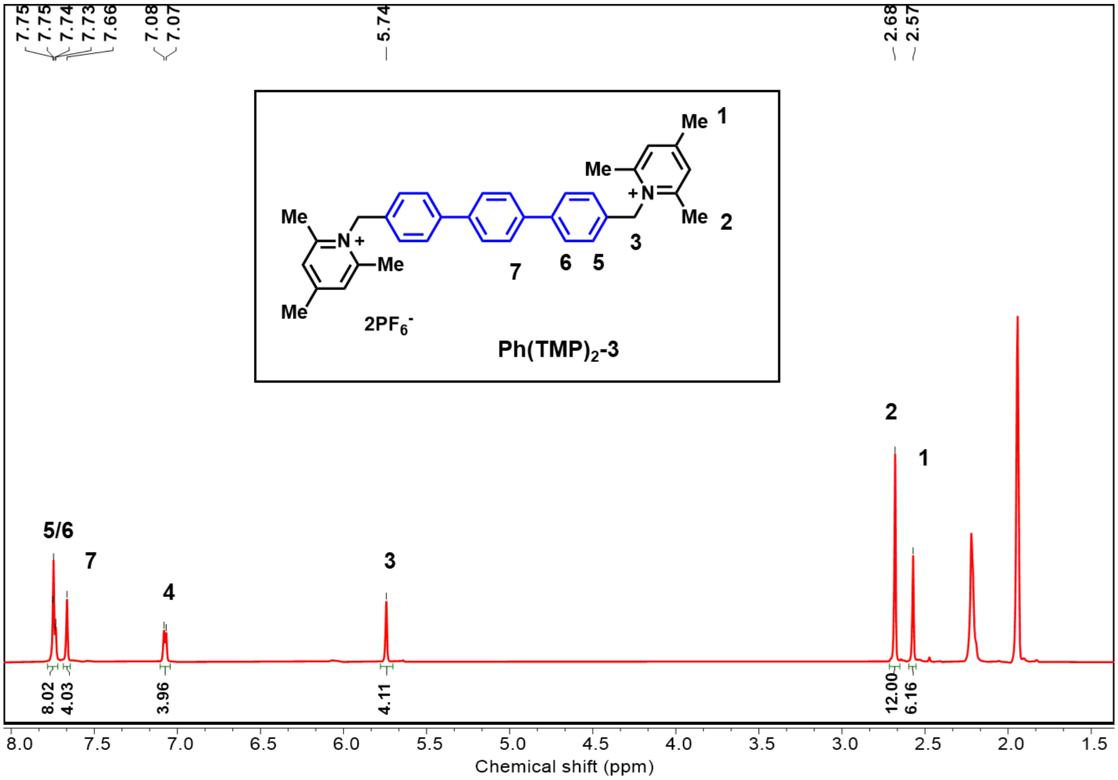

Supplementary Fig. 32 | ^{1}H NMR (600 MHz, Acetonitrile- d_3, 298 K) of Ph(TMP)$_2$–3.

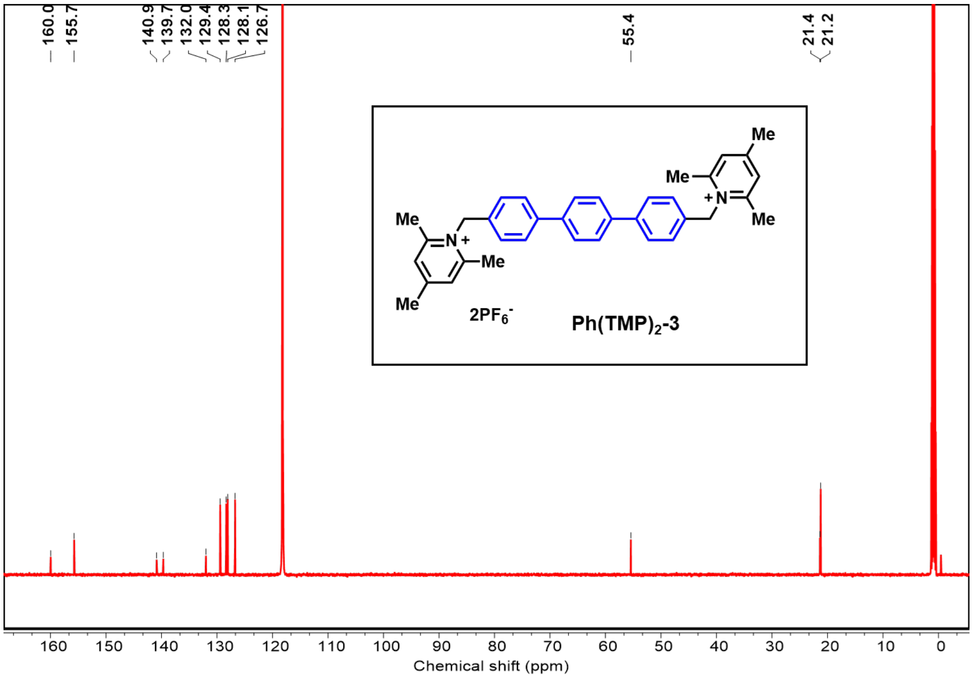

Supplementary Fig. 33 | ^{13}C NMR spectrum (151 MHz, Acetonitrile-d_3, 298 K) of Ph(TMP)$_2$-3.

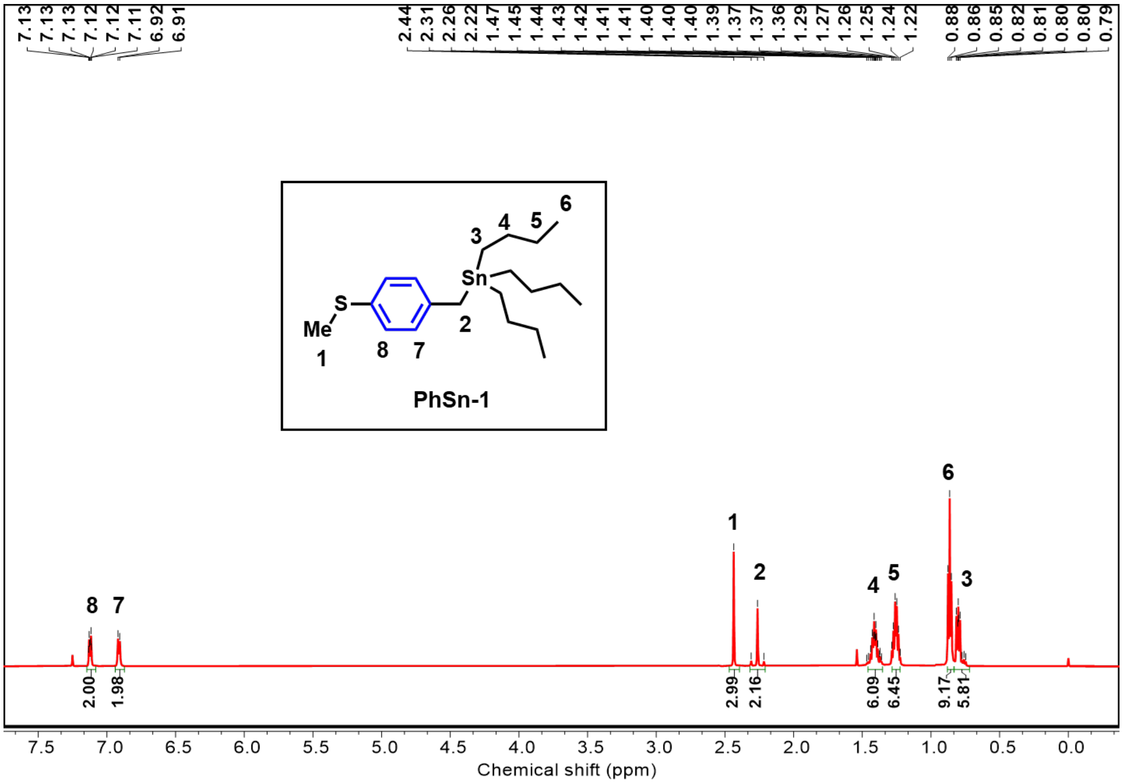

Supplementary Fig. 34 | ^{1}H NMR (600 MHz, Chloroform-*d*, 298 K) of PhSn–1.

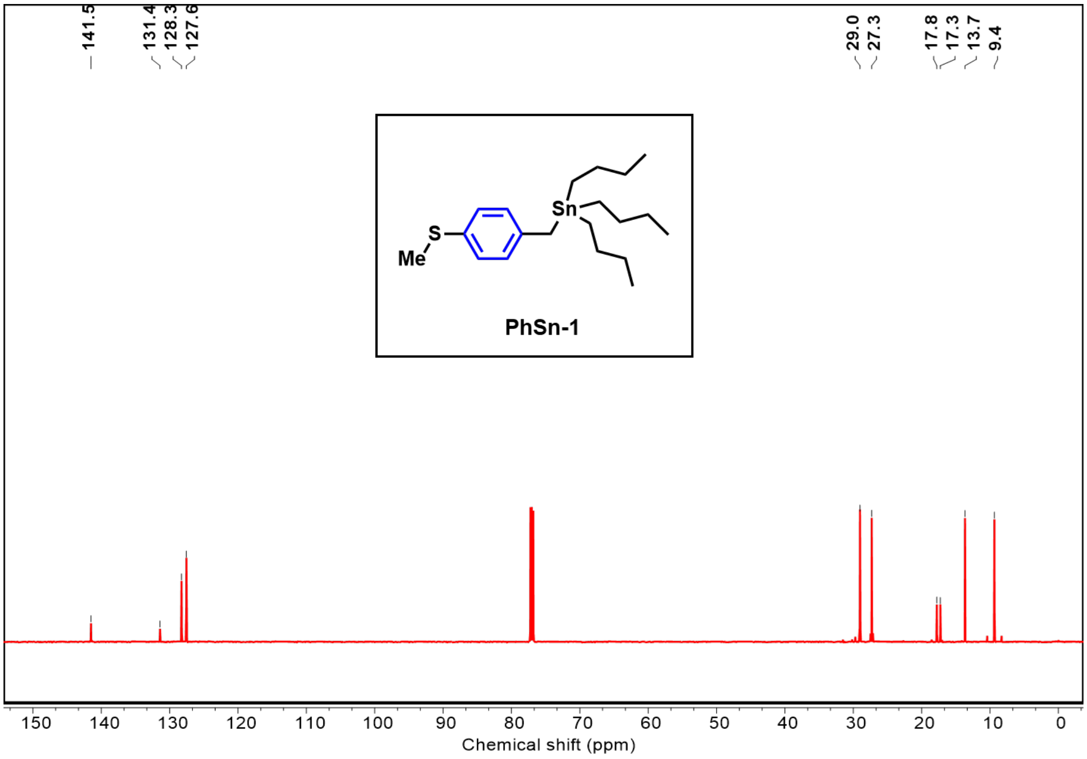

Supplementary Fig. 35 | ^{13}C NMR spectrum (151 MHz, Chloroform-*d*, 298 K) of PhSn–1.

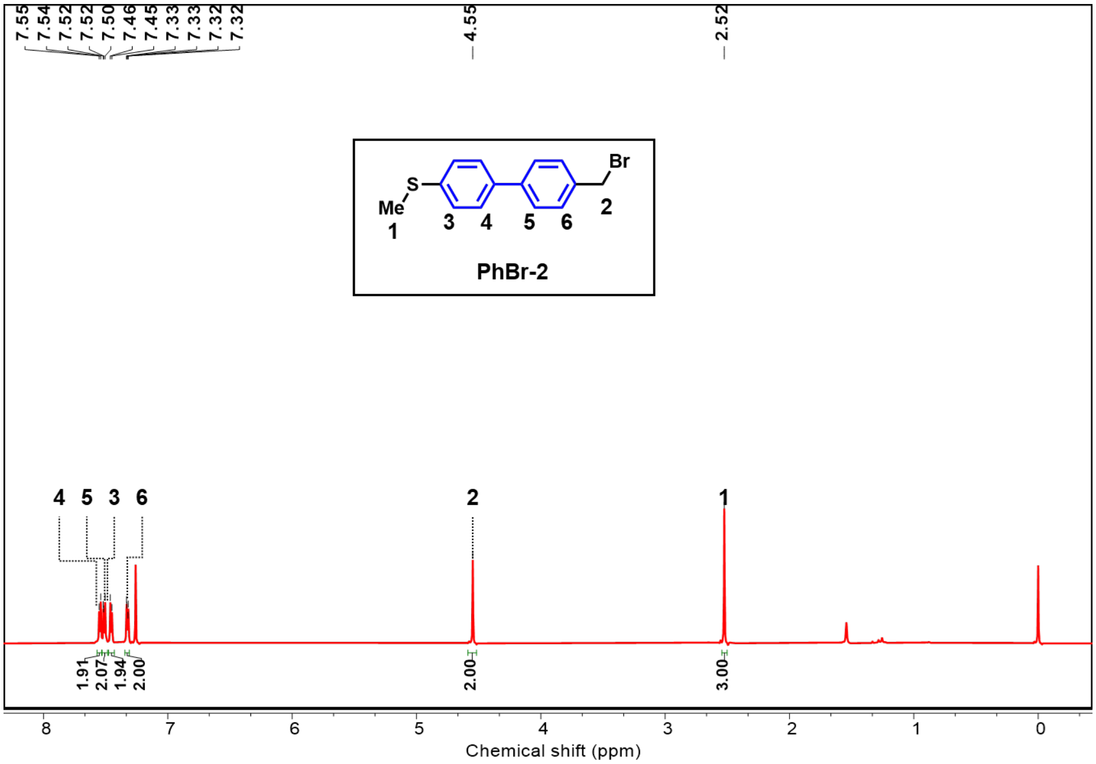

Supplementary Fig. 36 | ^{1}H NMR (600 MHz, Chloroform-*d*, 298 K) of PhBr–2.

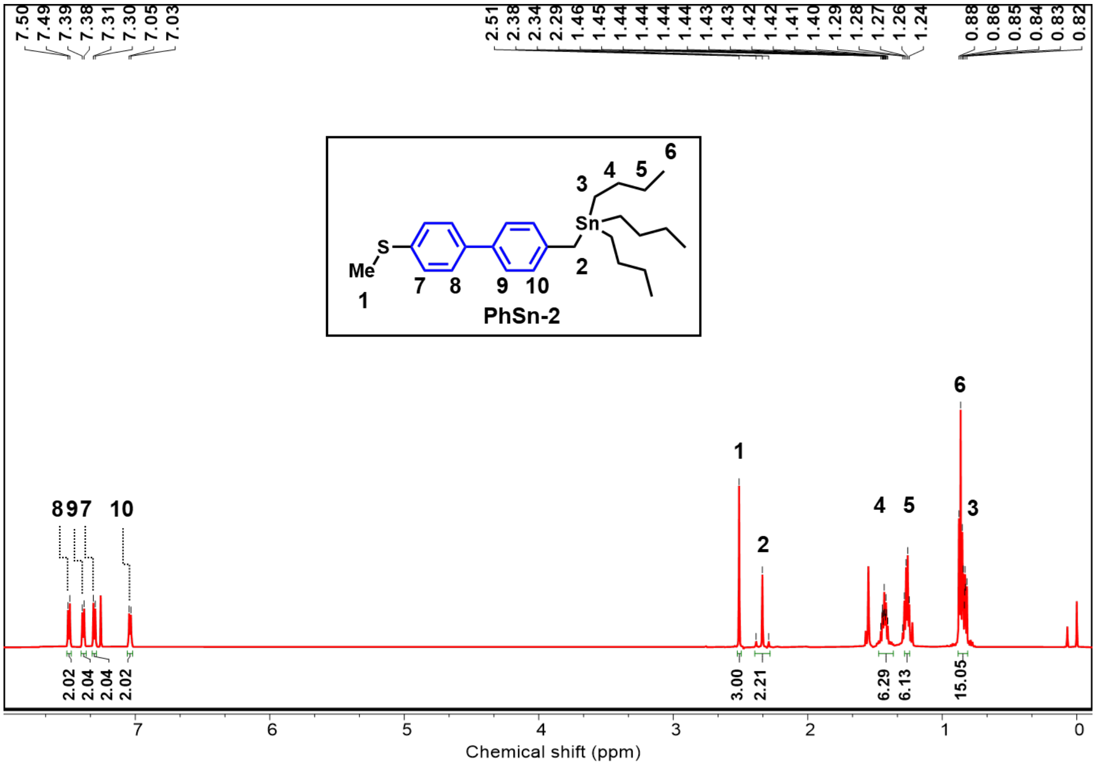

Supplementary Fig. 37 | ^{1}H NMR (600 MHz, Chloroform-*d*, 298 K) of PhSn–2.

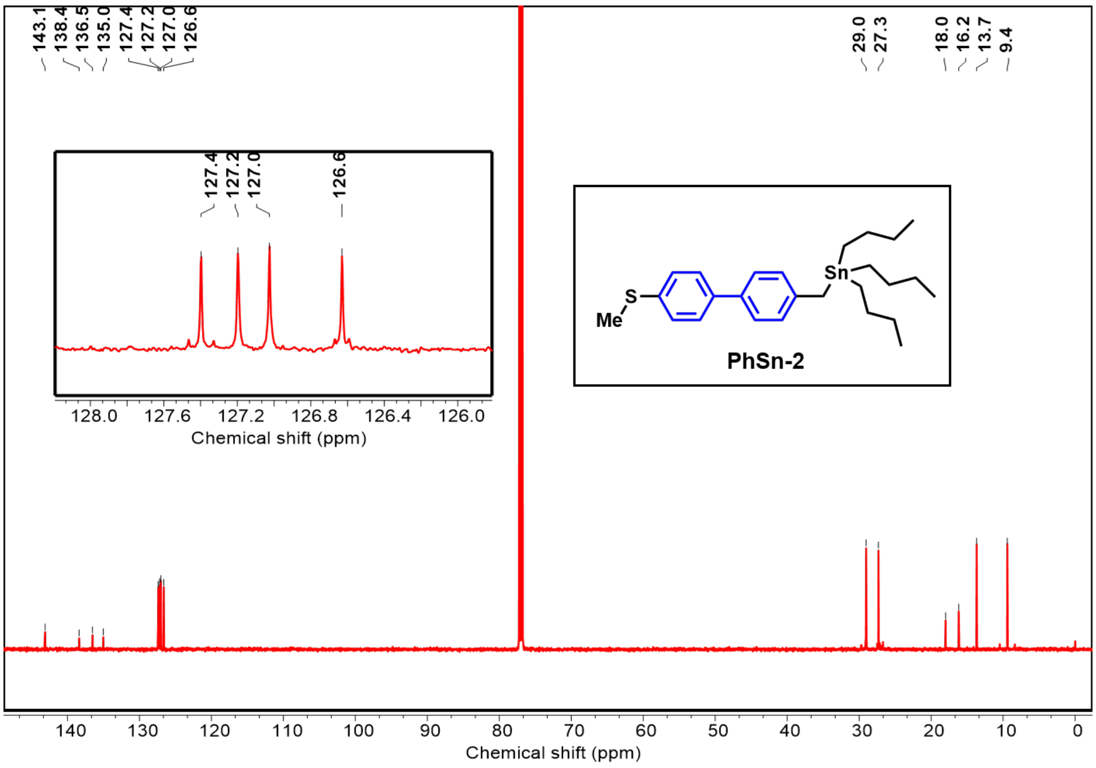

Supplementary Fig. 38 | ^{13}C NMR spectrum (151 MHz, Chloroform-*d*, 298 K) of PhSn–2.

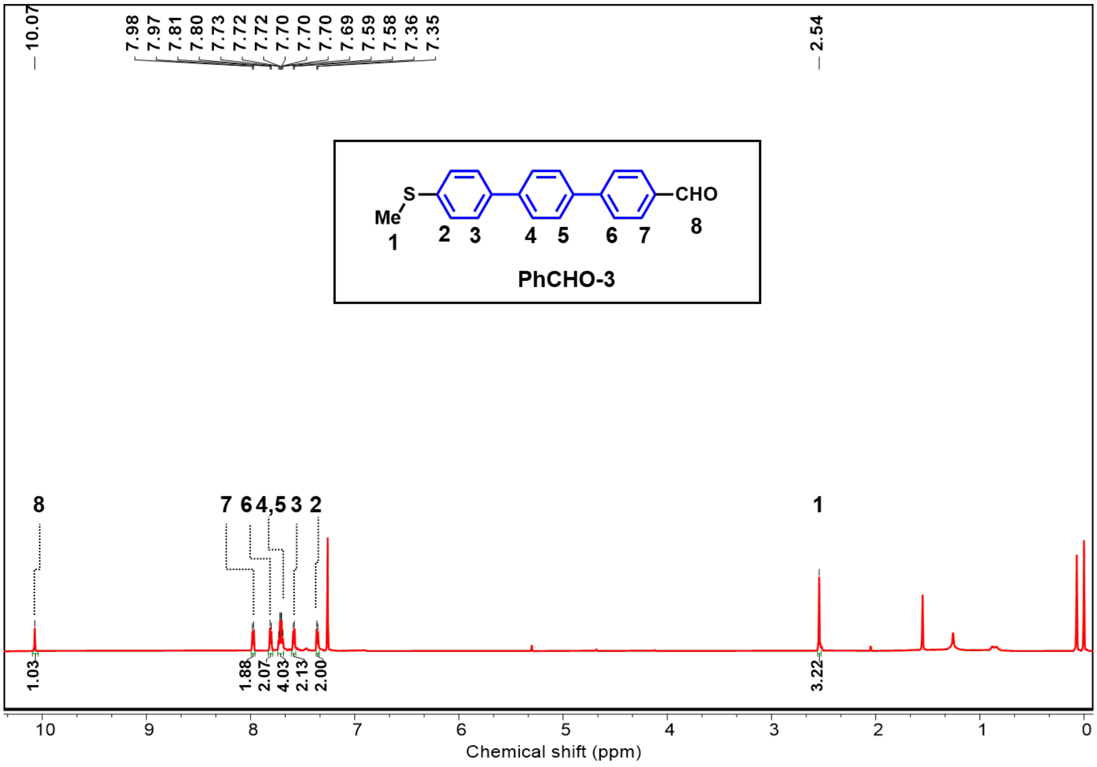

Supplementary Fig. 39 | ^{1}H NMR (600 MHz, Chloroform-*d*, 298 K) of PhCHO–3.

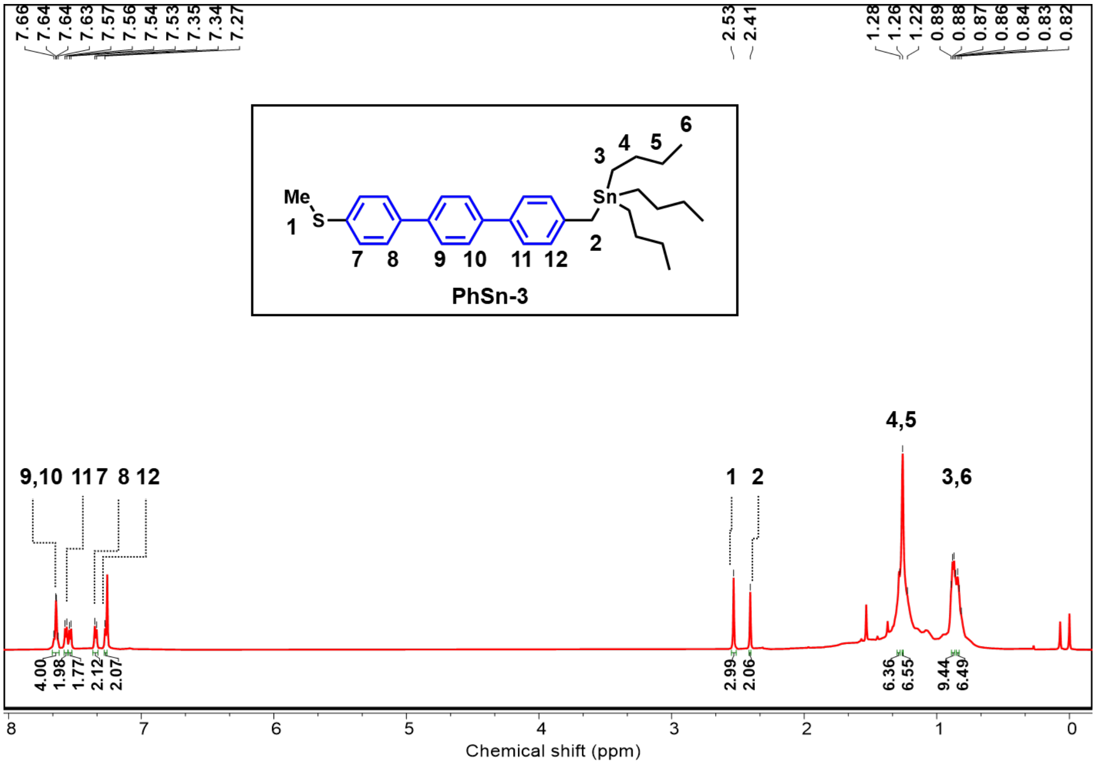

Supplementary Fig. 40 | ^{1}H NMR (600 MHz, Chloroform-*d*, 298 K) of PhSn–3.

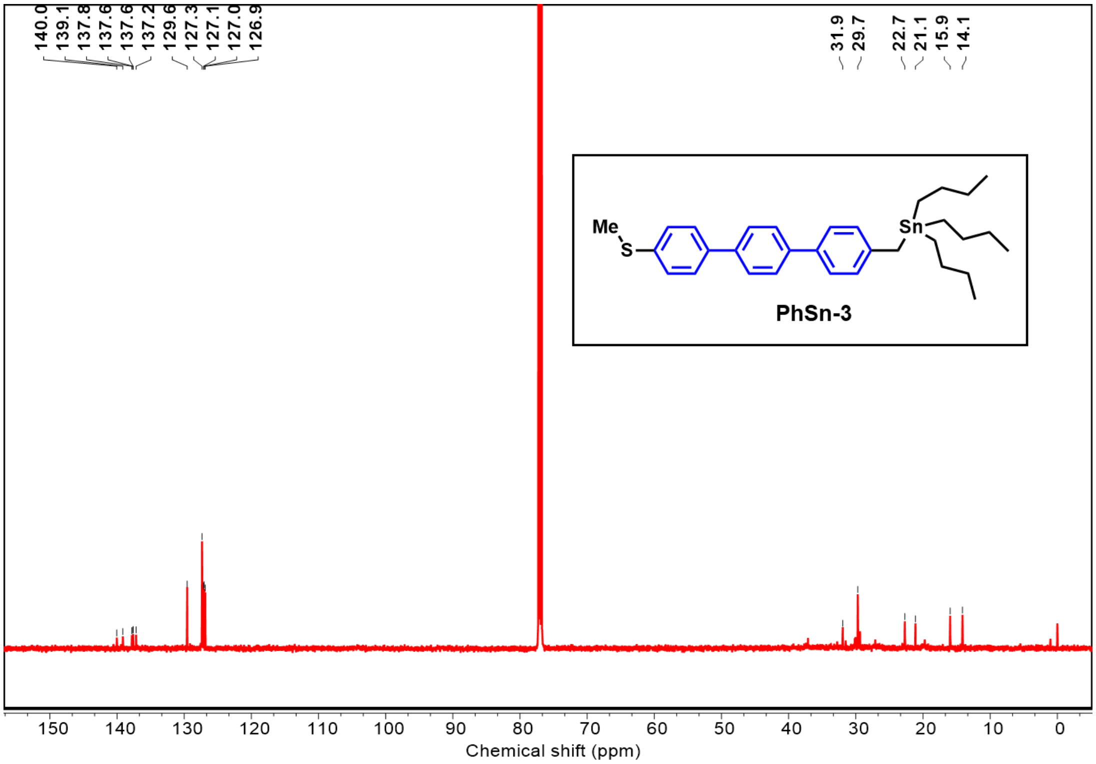

Supplementary Fig. 41 | ^{13}C NMR spectrum (151 MHz, Chloroform-*d*, 298 K) of PhSn–3.

Supplementary Note 3. Electrical data

We find that the conductance trends for both families are nearly linear on the plot, indicating that the conductance depends exponentially on length according to the relationship:

$$G = G_c \exp(-\beta L) \tag{1}$$

where G is the conductance, G_c is the contact conductance arising from the molecule−electrode contact, L is the length of the molecule, and β is the decay constant that depends on the tunneling barrier associated with the single-molecule junction. Utilizing **Eq. (1)**, we calculated (red dotted lines) a decay constant for PyBz–*n* of $\beta = 3.30 \pm 0.54$ nm^{-1}, a value that is comparable to the ex situ prepared Py–*n* family of 3.14±0.32 nm^{-1}. This phenomenon suggests the emergence of SET-induced formation of a new species, specifically Py–*n*, within the nanogaps.

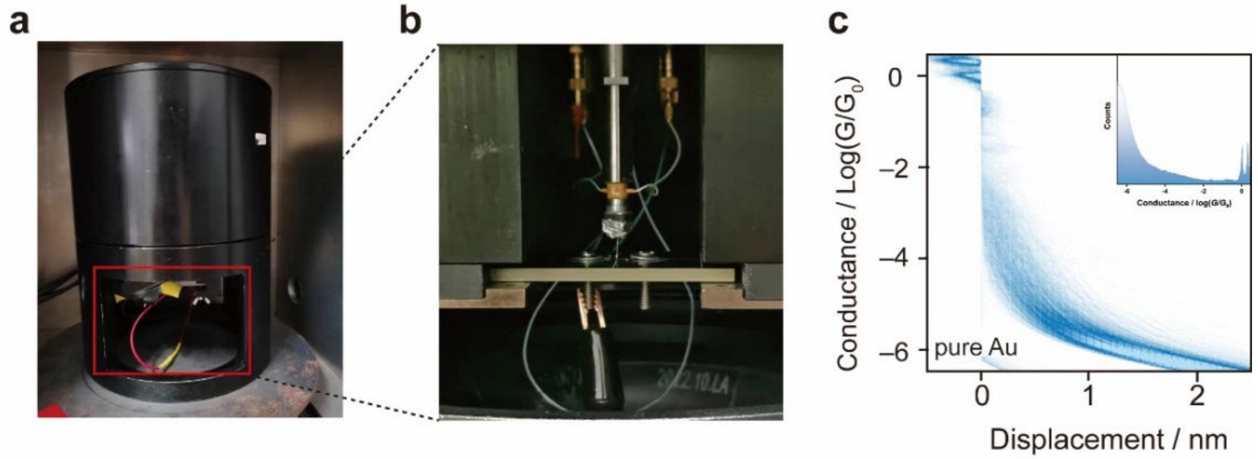

Supplementary Fig. 42 | The home-built STM-BJ set-up.[1] **a**, Photographs of the home-built STM set-up. **b**, An enlarged area of the tip and substrate. **c**, 2D Conductance-displacement histograms of control experiments illustrate the background current on a clean Au substrate without targeted compounds. Source data are provided as a Source Data file.

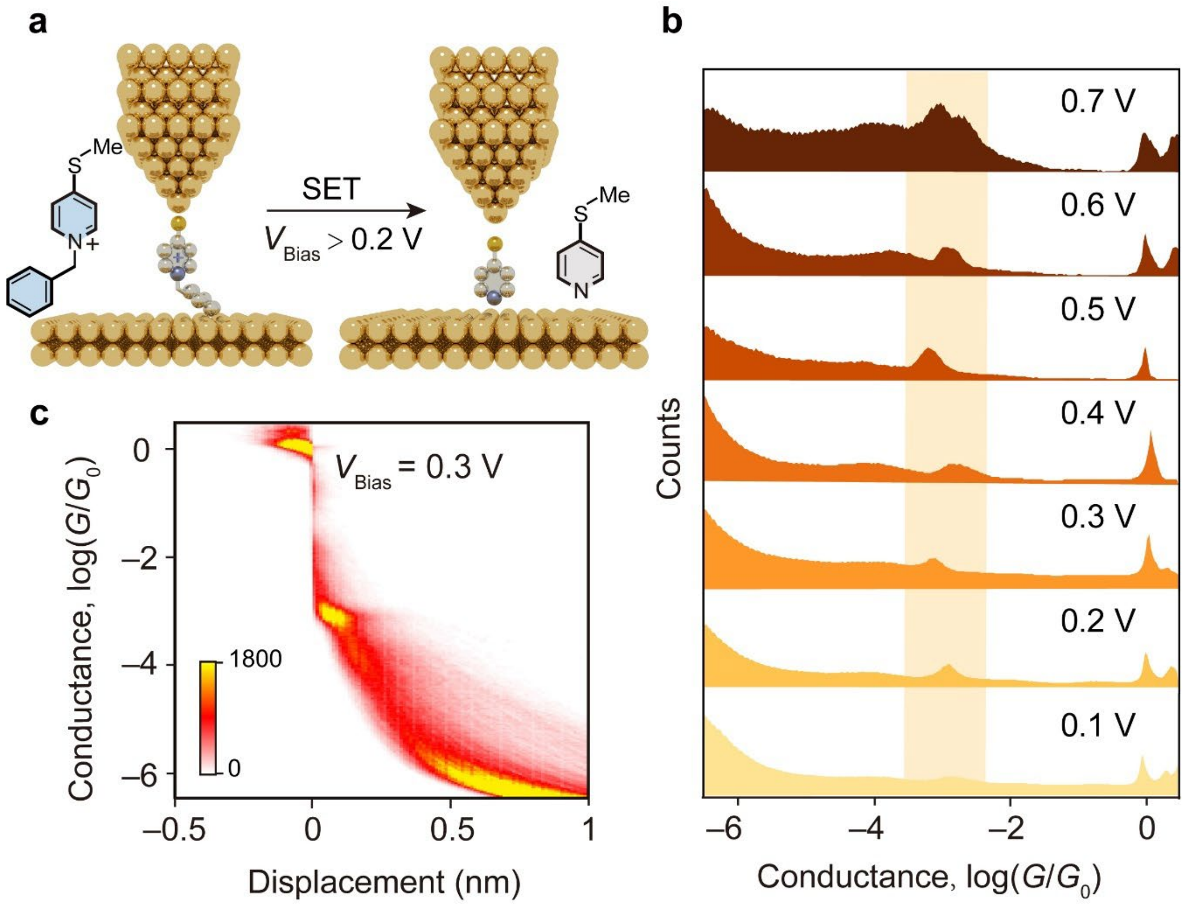

Supplementary Fig. 43 | Bias-dependent conductance measurements of PyBz–1. a, Scheme illustration showing the SET-induced transformation from PyBz–1 to Py–1. **b**, 1D conductance histograms showing the bias-dependent conductance signals at elevated bias voltages from 0.1 to 0.6 V. A dominant conductance peak appeared gradually at around $10^{-2.8}$ G_0 at a bias of 0.2 V, and its prominence increased as the bias voltage was raised up to 0.7 V. This phenomenon indicates the emergence of SET-induced formation of a new species, specifically Py–1, within the nanogaps. **c**, The typical 2D histogram of PyBz–1 at a bias voltage of 0.3 V. The colourbar in the figure represents the number of counts per 1000 traces in the 2D matrix plot. Source data are provided as a Source Data file.

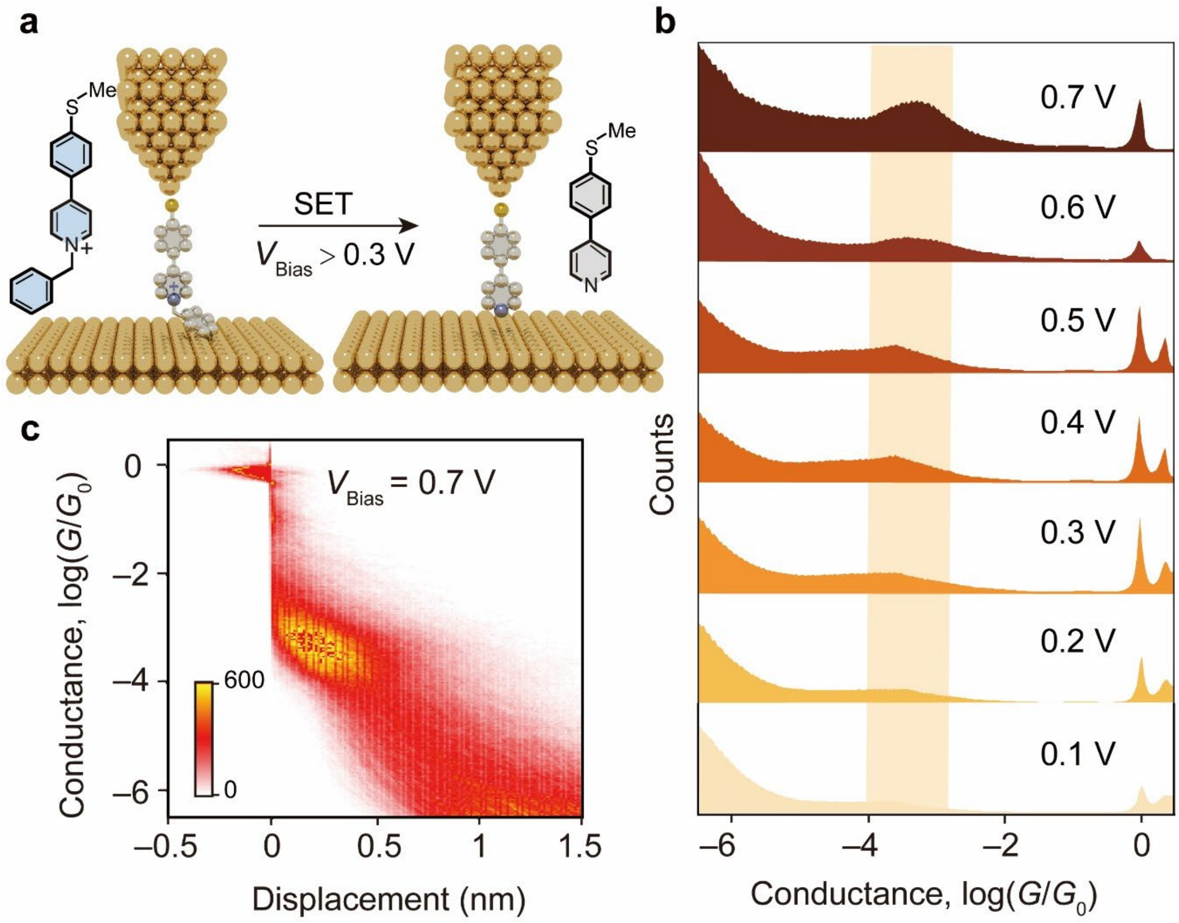

Supplementary Fig. 44 | Bias-dependent conductance measurements of PyBz–2. **a**, Scheme illustration showing the SET-induced transformation from PyBz–2 to Py–2. At increasing bias voltages, the 1D histogram from 0.1 V, 0.2 V, 0.3 V, 0.4 V, 0.5 V, 0.6 V to 0.7 V were shown in **b**. A conductance peak appeared gradually at around $10^{-3.2}$ G_0 at a bias of 0.3 V, and its prominence increased as the bias voltage was raised up to 0.7 V. This phenomenon indicates the emergence of SET-induced formation of a new species, specifically Py–2, within the nanogaps. **c**, The typical 2D histogram of PyBz–2 at a bias voltage of 0.7 V. The colourbar in the figure represents the number of counts per 1000 traces in the 2D matrix plot. Source data are provided as a Source Data file.

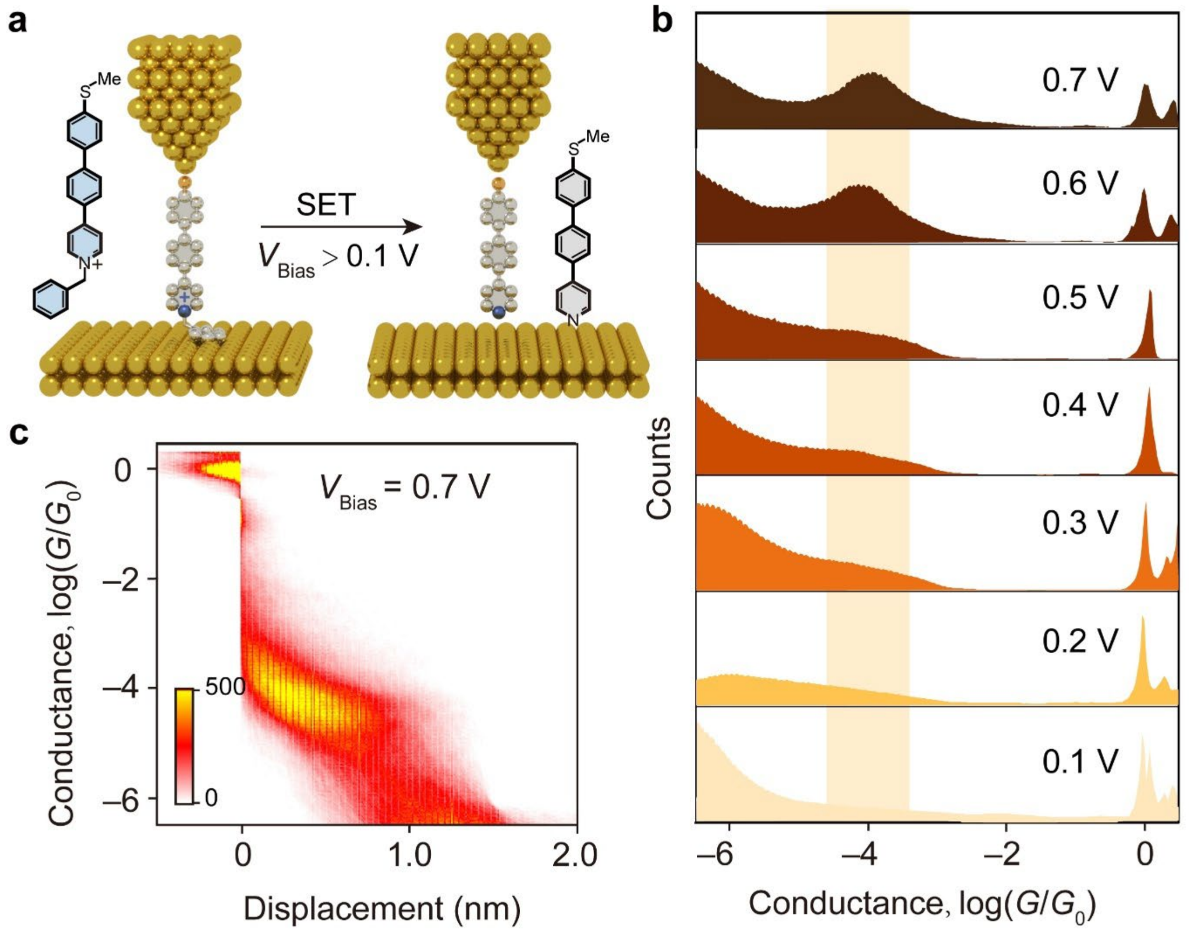

Supplementary Fig. 45 | Bias-dependent conductance measurements of PyBz–3. a, Scheme illustration showing the SET-induced transformation from PyBz–3 to Py–3. At increasing bias voltages**,** the 1D histogram from 0.1 V, 0.2 V, 0.3 V, 0.4 V, 0.5 V, 0.6 V to 0.7 V were shown in **b**. A conductance peak appeared gradually at around $10^{-4.0}$ G_0 at a bias of 0.4 V, and its prominence increased as the bias voltage was raised up to 0.7 V. This phenomenon indicates the emergence of SET-induced formation of a new species, specifically Py–3, within the nanogaps. **c**, The typical 2D histogram of PyBz–3 at a bias voltage of 0.7 V. The colourbar in the figure represents the number of counts per 1000 traces in the 2D matrix plot. Source data are provided as a Source Data file.

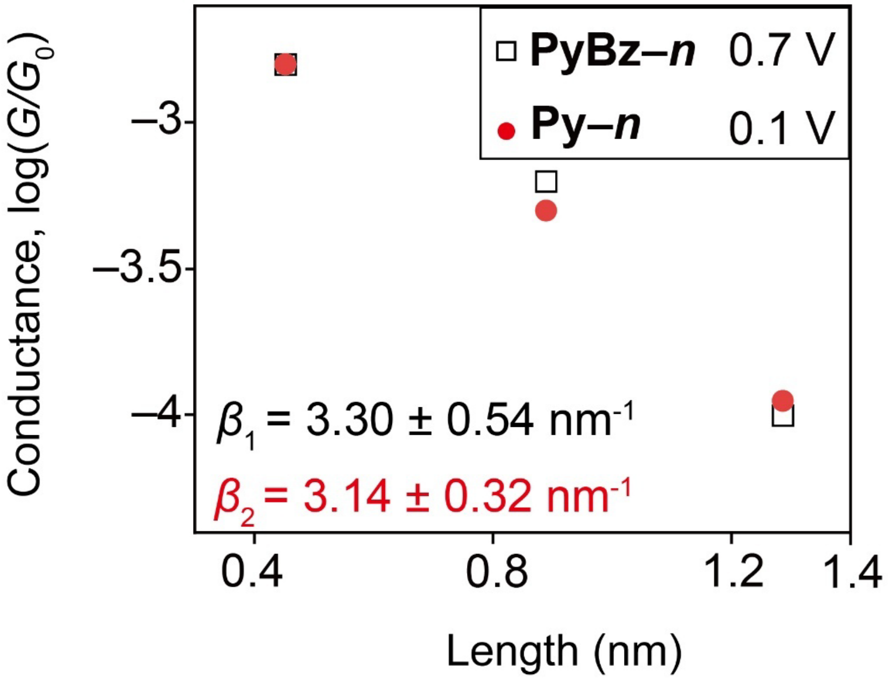

Supplementary Fig. 46 | Overview of the conductance measurements. The experimental values of conductance were extracted from the one-dimensional Gaussian-fitted conductance of PyBz–*n* under a high bias voltage of 0.7 V (blue dots) and Py–*n* prepared ex situ and measured at a bias voltage of 0.1 V (cyan dots). Source data are provided as a Source Data file.

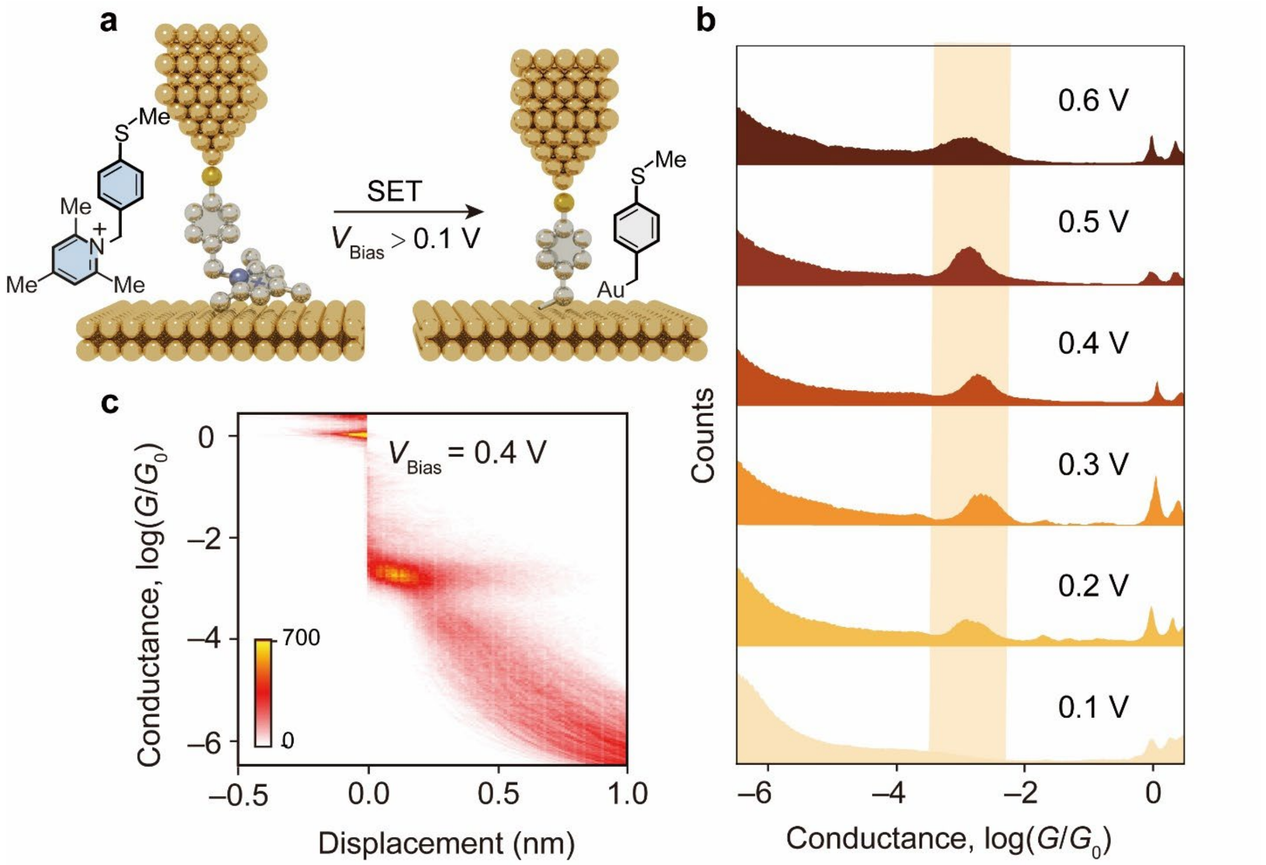

Supplementary Fig. 47 | Bias-dependent conductance measurements of PhTMP–1. **a**, Scheme illustration showing the SET-induced transformation from PhTMP–1 to Au–S-Ph[1]-C–Au junction. **b**, 1D conductance histograms showing the bias-dependent conductance signals at elevated bias voltages from 0.1 to 0.6 V. A dominant conductance peak appeared gradually at around $10^{-2.9}$ G_0 at a bias of 0.2 V, and its prominence increased as the bias voltage was raised up to 0.6 V. This phenomenon indicates the emergence of SET-induced formation of a new species, specifically Ph–1•, within the nanogaps. **c**, The typical 2D histogram obtained at a bias voltage of 0.4 V. The colourbar in the figure represents the number of counts per 1000 traces in the 2D matrix plot. Source data are provided as a Source Data file.

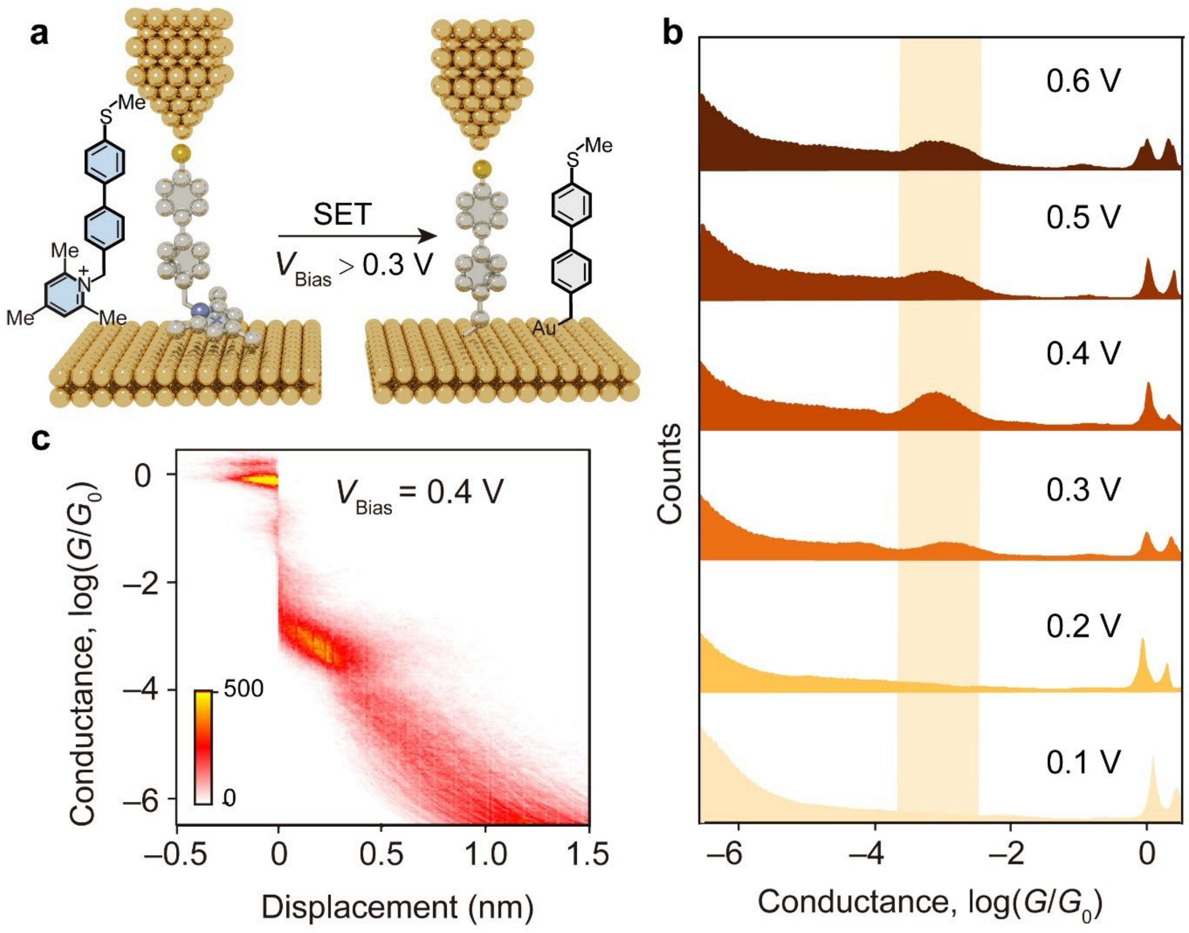

Supplementary Fig. 48 | Bias-dependent conductance measurement of PhTMP–2. a, Scheme illustration showing the SET-induced transformation from PhTMP–2 to Au–S-Ph^2-C–Au junction. **b**, 1D conductance histograms showing the bias-dependent conductance signals at elevated bias voltages from 0.1 to 0.6 V. A dominant conductance peak appeared gradually at around $10^{-3.2}$ G_0 at a bias of 0.3 V, and its prominence increased as the bias voltage was raised up to 0.6 V. This phenomenon indicates the emergence of SET-induced formation of a new species, specifically Ph–2$^•$, within the nanogaps. **c**, The typical 2D histogram obtained at a bias voltage of 0.4 V. The colourbar in the figure represents the number of counts per 1000 traces in the 2D matrix plot. Source data are provided as a Source Data file.

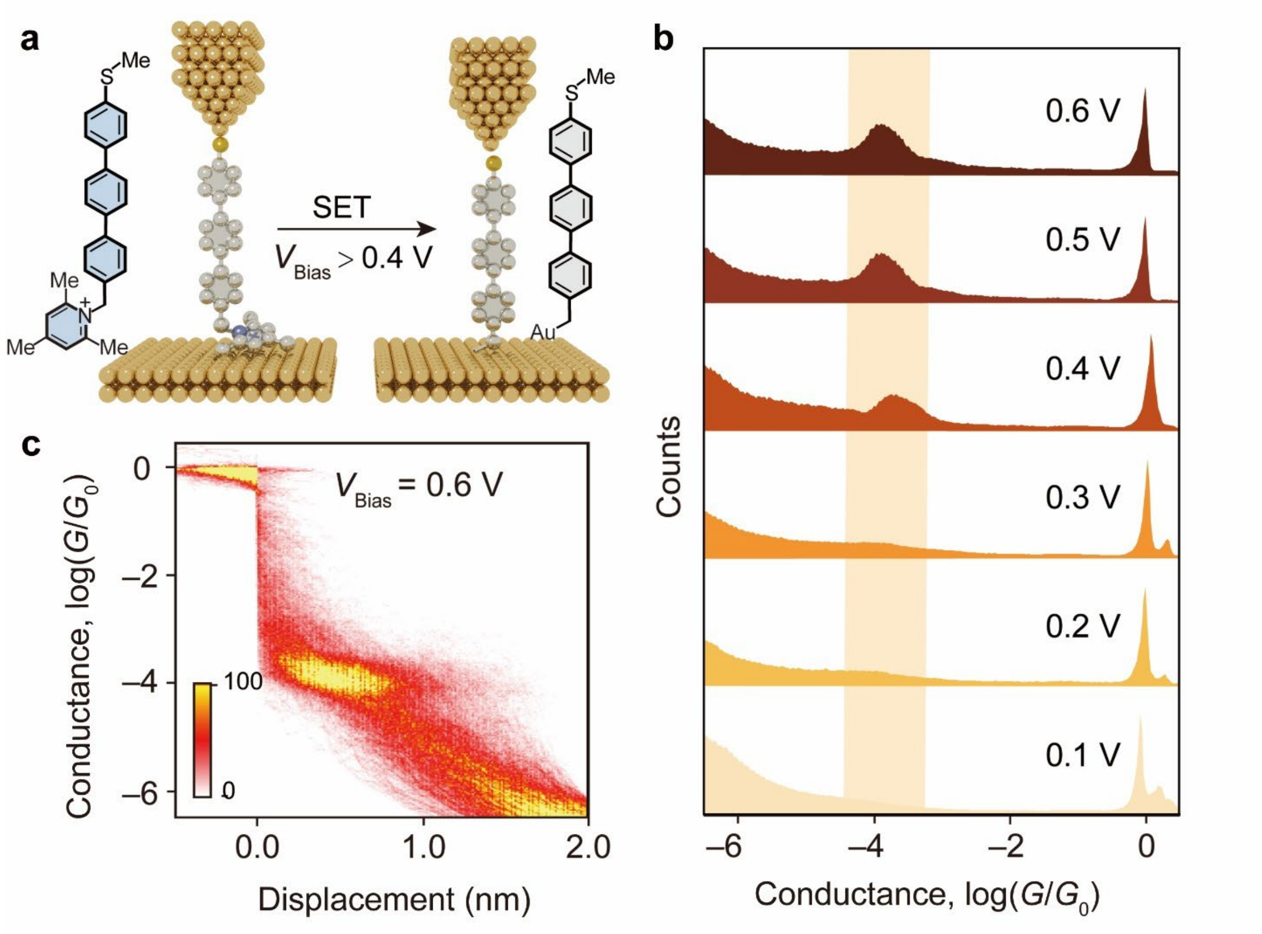

Supplementary Fig. 49 | Bias-dependent conductance measurement of PhTMP–3. **a**, Scheme illustration showing the SET-induced transformation from PhTMP–3 to Au–S-Ph^3-C–Au junction. **b**, 1D conductance histograms showing the bias-dependent conductance signals at elevated bias voltages from 0.1 to 0.6 V. A dominant conductance peak appeared gradually at around $10^{-3.9}$ G_0 at a bias of 0.4 V, and its prominence increased as the bias voltage was raised up to 0.6 V. This phenomenon indicates the emergence of SET-induced formation of a new species, specifically Ph–3$^{\bullet}$, within the nanogaps. **c**, The typical 2D histogram obtained at a bias voltage of 0.6 V. The colourbar in the figure represents the number of counts per 1000 traces in the 2D matrix plot. Source data are provided as a Source Data file.

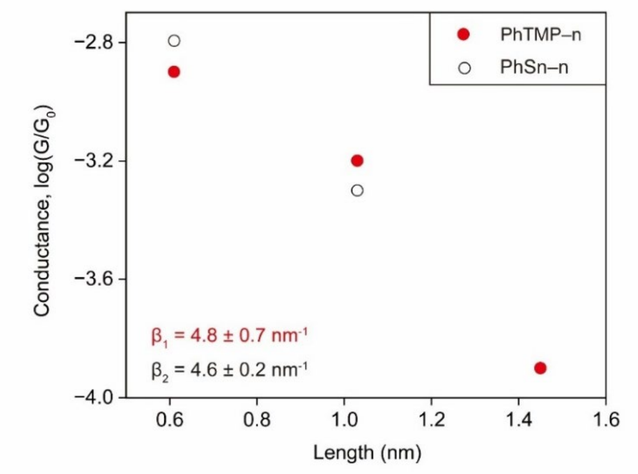

Supplementary Fig. 50 | Overview of the conductance measurements. The experimental values of conductance were extracted from the one-dimensional Gaussian-fitted conductance of PhTMP–*n* under a high bias voltage of 0.6 V (black square) and the β value is 4.8 ± 0.7 nm^{-1} PhSn–*n* measured at a bias voltage of 0.1 V (red dots) and the β value is 4.6 ± 0.2 nm^{-1}. Source data are provided as a Source Data file.

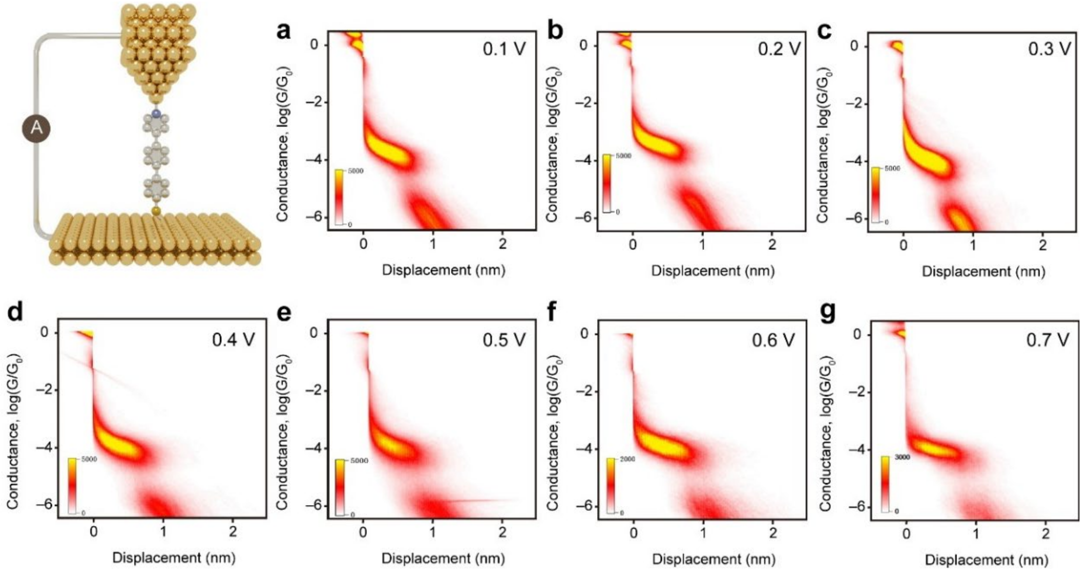

Supplementary Fig. 51 | Bias-dependent measurements of ex situ-prepared Py–3. a–**g**, 2D conductance-displacement histograms obtained at different bias voltages starting from **a**, 0.1 V, **b**, 0.2 V, **c**, 0.3 V, **d**, 0.4 V, **e**, 0.5 V, **f**, 0.6 V, and **g**, 0.7 V. The colourbar in the figure represents the number of counts per 1000 traces in the 2D matrix plot. Source data are provided as a Source Data file.

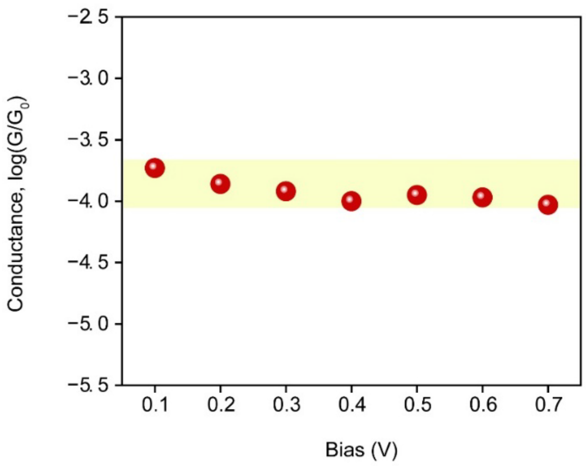

Supplementary Fig. 52 | Bias-dependent experimental conductance statistics of ex situ-prepared Py–3. The results show no dependence of conductance on bias voltage. Source data are provided as a Source Data file.

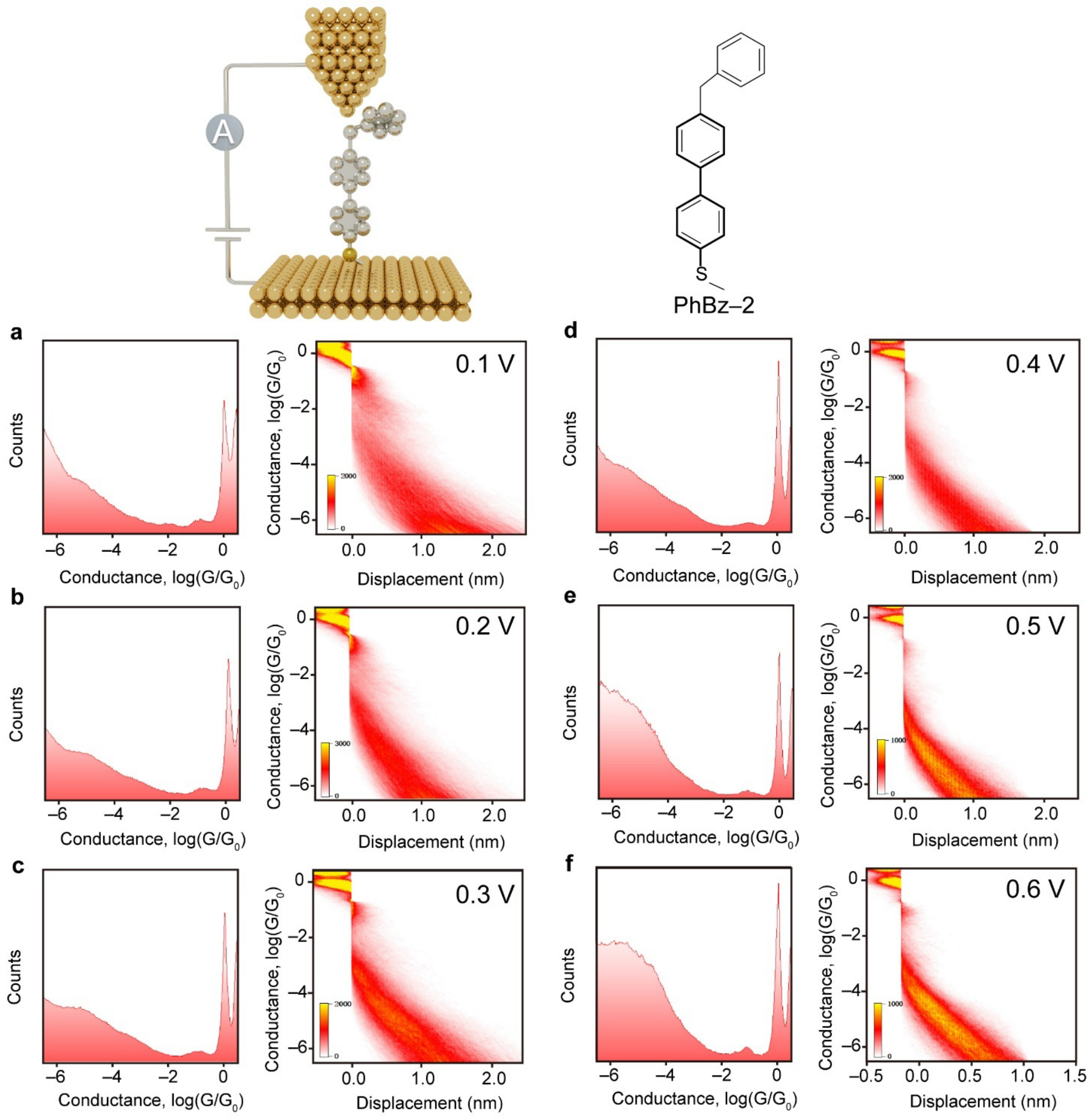

Supplementary Fig. 53 | Bias-dependent conductance measurement of PhBz–2 a–**f**, The 2D conductance-displacement histograms obtained at different bias voltages starting from **a**, 0.1 V, **b**, 0.2 V, **c**, 0.3 V, **d**, 0.4 V, **e**, 0.5 V, and **f**, 0.6 V. The colourbar in the figure represents the number of counts per 1000 traces in the 2D matrix plot. Source data are provided as a Source Data file.

Supplementary Note 4. Characterization of radicals

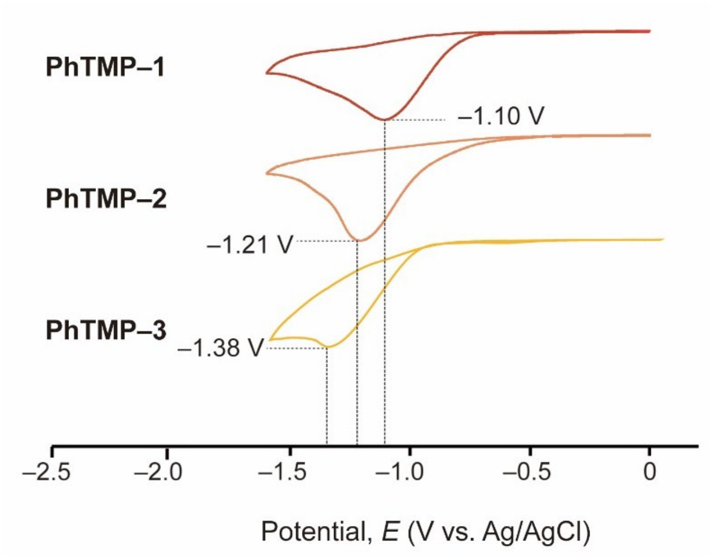

Supplementary Fig. 54 | Cyclic voltammograms (CVs) experiments performed on PhTMP–*n*•BF$_4$ (*n* = 1, 2, and 3). The CV experiments were performed in MeCN with potassium tetrafluoroborate (KBF$_4$, 10 mM) as the auxiliary electrolyte. Working electrode (WE): glassy carbon; Reference electrode (RE): Ag/AgCl; Counter electrode (CE): Pt wire. Source data are provided as a Source Data file.

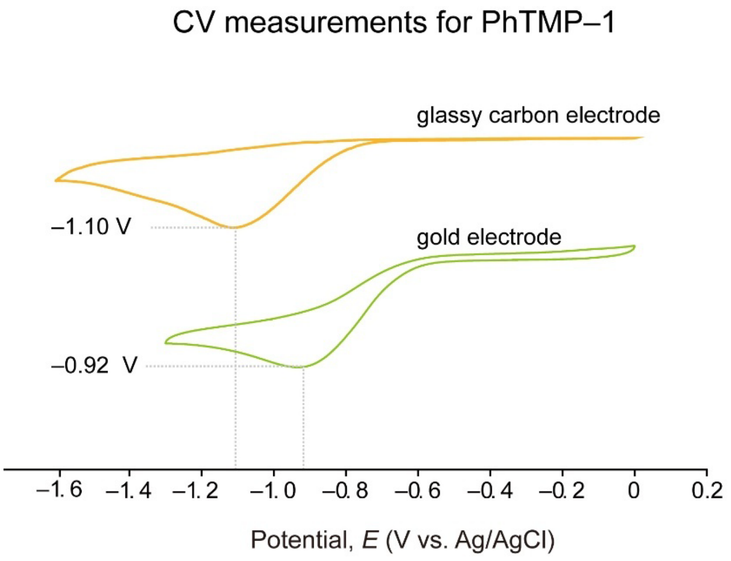

Supplementary Fig. 55 | Cyclic voltammograms (CV) experiments performed on PhTMP–

1•BF_4. The CV experiments were performed in MeCN with potassium tetrafluoroborate (KBF_4, 10 mM) as the auxiliary electrolyte. Working electrode (WE): Au; Reference electrode (RE): Ag/AgCl; Counter electrode (CE): Pt wire. When the Au disk electrode is used as the working electrode, the reduction potential of PhTMP–1 is approximately –0.9 V, which is shifted by 0.2 V compared to the potential obtained by glassy carbon electrode. We believe that the adsorption of the Au electrode on the molecules facilitates electron transfer, thus lowering the reduction potential. Source data are provided as a Source Data file.

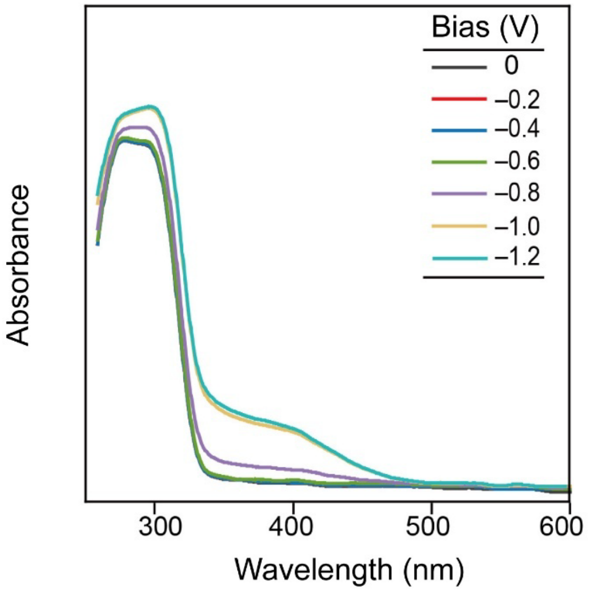

Supplementary Fig. 56 | UV-Vis spectroelectrochemical experiments performed on PhTMP–2•BF_4. The Ph–2$^{•}$ radical species was generated through electrochemical reduction at various potentials in a MeCN solution containing 1 mM of PhTMP–2•BF_4. Source data are provided as a Source Data file.

Supplementary Note 5. Theoretical calculations

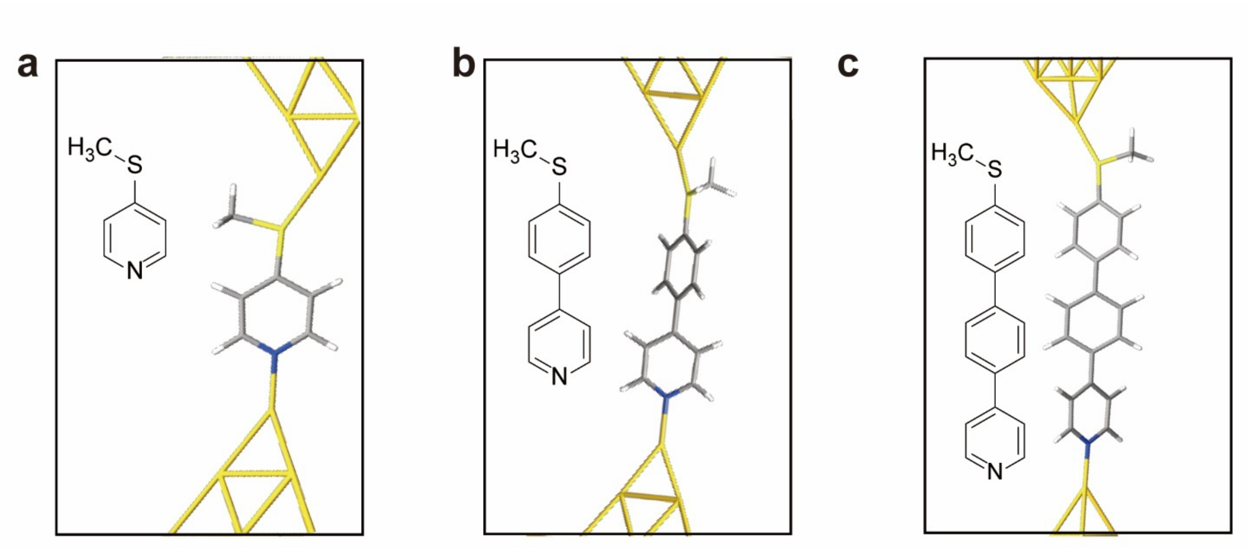

Supplementary Fig. 57 | Optimized junction geometries for Au–S-Py^n-N–Au. Optimized junction geometries for **a**, Py–1, **b**, Py–2, and **c**, Py–3 show Au top electrodes binding to thiolmethyl anchors and bottom electrodes binding to pyridine anchors.

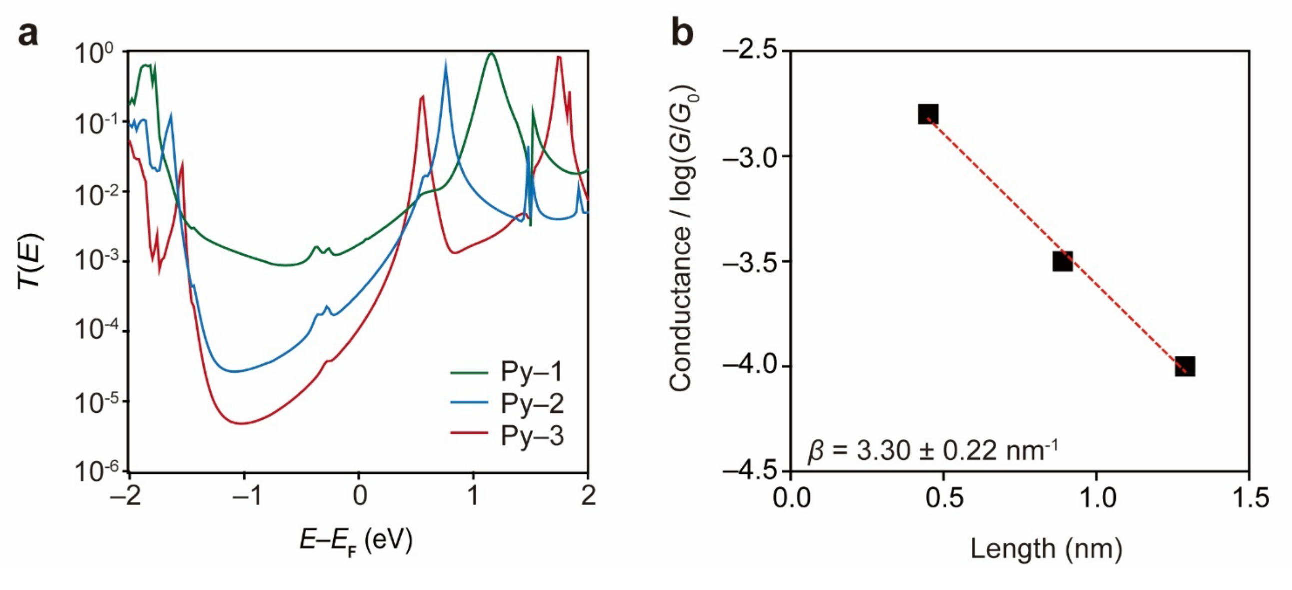

Supplementary Fig. 58 | DFT calculated transmission spectra for the junctions Au–S-Py^n-N–Au. **a**, The calculated transmission functions for Py–1, Py–2 and Py–3. **b**, The decay constants obtained by theoretical calculations. Source data are provided as a Source Data file.

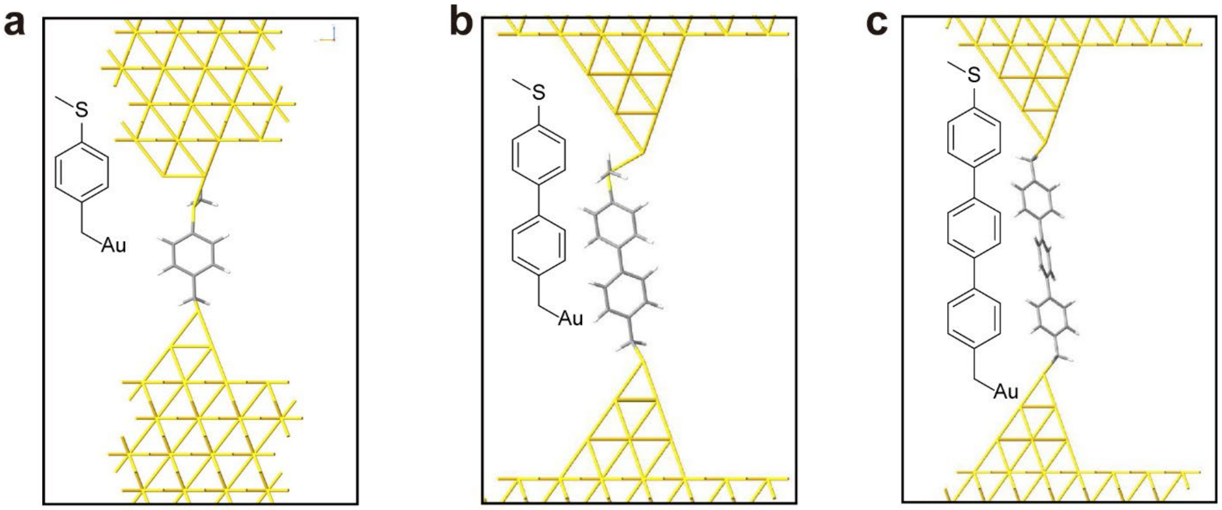

Supplementary Fig. 59 | Optimized junction geometries for Au–S-Ph^n-C–Au junction. Optimized junction geometries for **a**, Au–S-Ph^1-C–Au junction, **b**, Au–S-Ph^2-C–Au junction, and **c**, Au–S-Ph^3-C–Au junction demonstrate Au top electrodes binding to thiolmethyl anchors and bottom electrodes binding to C atom forming Au–C covalent contacts.

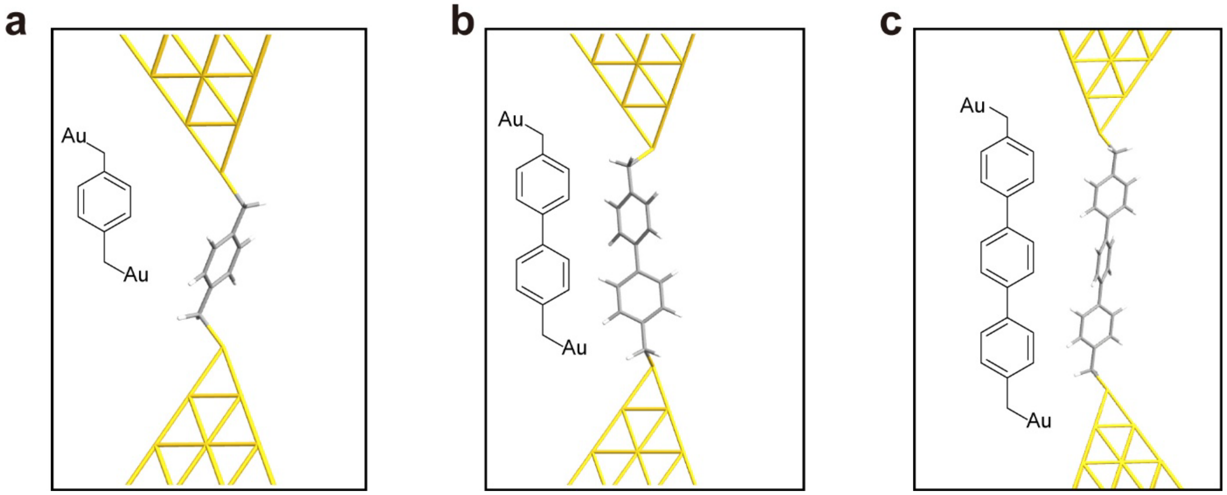

Supplementary Fig. 60 | Optimized junction geometries for Au–C-Ph^n-C–Au junction. Optimized junction geometries for **a**, Au–C-Ph^1-C–Au junction, **b**, Au–C-Ph^2-C–Au junction, and **c**, Au–C-Ph^3-C–Au junction demonstrate Au top electrode bottom electrodes binding to C atom forming Au–C covalent contacts.

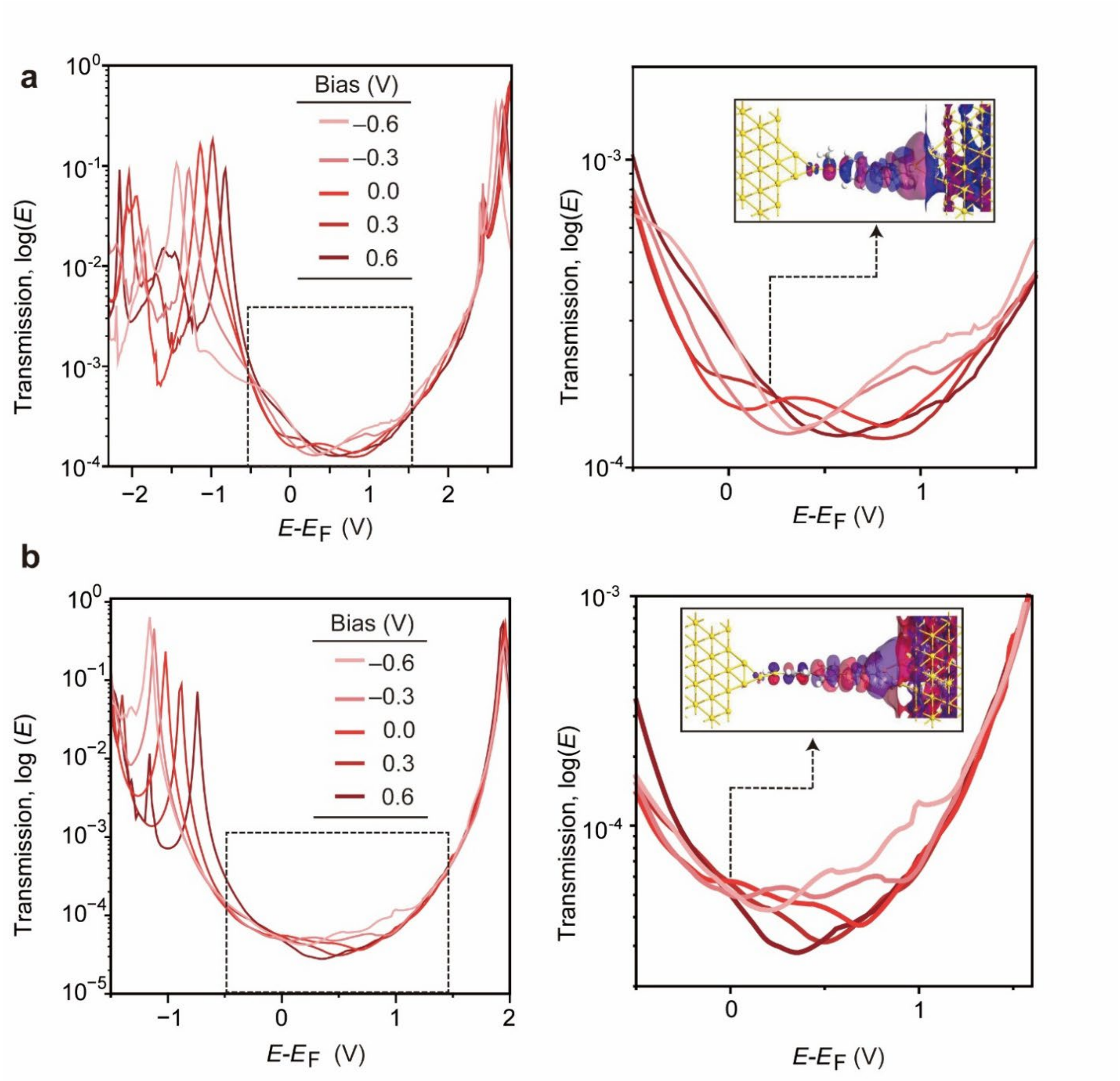

Supplementary Fig. 61 | DFT calculated transmission spectra for the junctions Au–S-Ph^n-C–Au junctions. a, DFT calculated transmission spectra for Au–S-Ph^2-C–Au junction at −0.6 V (light red) to +0.6 V (dark red). Right: Enlarge the portion within the dashed box. Right insert: The arrow indicates the position of transmission eigenstate corresponding to gold–carbon gateway state at 0.2 eV. **b**, DFT calculated transmission spectra for Au–S-Ph^3-C–Au junction at −0.6 V (light red) to +0.6 V (dark red). Right: Enlarge the portion within the dashed box. Right insert: The arrow indicates the position of transmission eigenstate corresponding to gold–carbon gateway state at 0 eV. Source data are provided as a Source Data file.

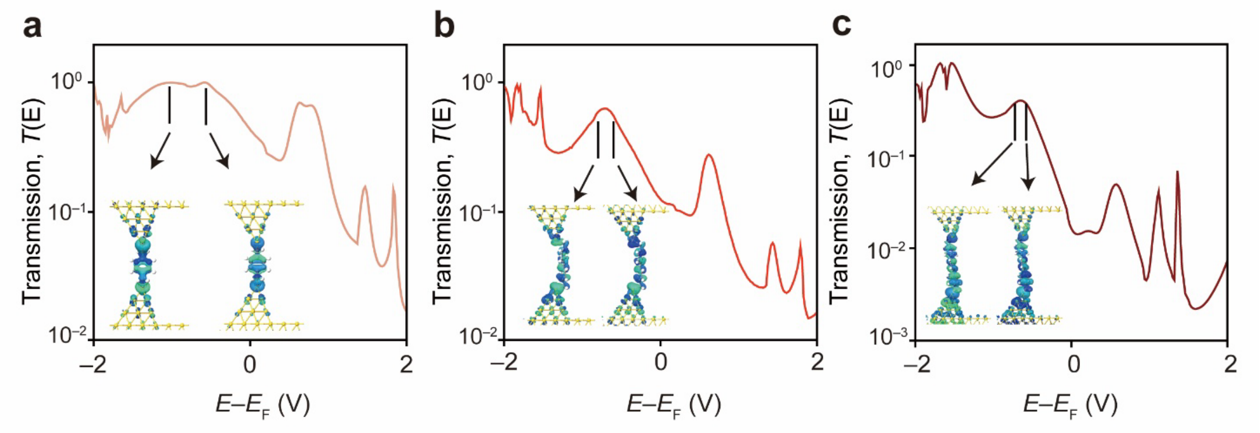

Supplementary Fig. 62 | DFT calculated transmission spectra for Au–C-Ph^{n}-C–Au junctions. **a**, DFT calculated transmission spectrum for the Au–C-Ph^{1}-C–Au junction. Insert: The arrow indicates the position of transmission eigenstate corresponding to Au–C gateway state at −0.56 and −1.02 eV. **b**, DFT calculated transmission spectrum for the Au–C-Ph^{2}-C–Au junction. Insert: The arrow indicates the position of transmission eigenstate corresponding to Au–C gateway state at −0.6 and −0.8 eV. **c**, DFT calculated transmission spectrum for the Au–C-Ph^{3}-C–Au junction. Insert: The arrow indicates the position of transmission eigenstate corresponding to Au–C gateway state at −0.62 and −0.7 eV. Source data are provided as a Source Data file.

Supplemental references

1 Kaliginedi, V. et al. Correlations between molecular structure and single-junction conductance: a case study with oligo(phenylene-ethynylene)-type wires. *J. Am. Chem. Soc.* **134**, 5262–5275 (2012).

2 Manrique, D. Z. et al. A quantum circuit rule for interference effects in single-molecule electrical junctions. *Nat. Commun.* **6**, 6389 (2015).

3 Ferrer, J. et al. GOLLUM: A next-generation simulation tool for electron, thermal and spin transport. *New J. Phys.* **16**, 093029 (2014).
